# Genetic mapping of canine fear and aggression

**DOI:** 10.1186/s12864-016-2936-3

**Published:** 2016-08-08

**Authors:** Isain Zapata, James A. Serpell, Carlos E. Alvarez

**Affiliations:** 1 Center for Molecular and Human Genetics, The Research Institute at Nationwide Children’s Hospital, Columbus, OH 43205 USA; 2 Center for the Interaction of Animals and Society, School of Veterinary Medicine, University of Pennsylvania, Philadelphia, PA 19104 USA; 3 Department of Pediatrics, The Ohio State University College of Medicine, Columbus, OH 43210 USA; 4 Department of Veterinary Clinical Sciences, The Ohio State University College of Veterinary Medicine, Columbus, OH 43210 USA

**Keywords:** Canine, Behavior, Emotions, Fear, Aggression, Sociability, Morphology, GWAS, SNP, Mapping, C-BARQ, IGSF1, GNAT3, CD36, IGF1, HMGA2, HS6ST2, Hypothalamic-pituitary-adrenal axis, HPA

## Abstract

**Background:**

Fear/anxiety and anger/aggression greatly influence health, quality of life and social interactions. They are a huge burden to wellbeing, and personal and public economics. However, while much is known about the physiology and neuroanatomy of such emotions, little is known about their genetics – most importantly, why some individuals are more susceptible to pathology under stress.

**Results:**

We conducted genomewide association (GWA) mapping of breed stereotypes for many fear and aggression traits across several hundred dogs from diverse breeds. We confirmed those findings using GWA in a second cohort of partially overlapping breeds. Lastly, we used the validated loci to create a model that effectively predicted fear and aggression stereotypes in a third group of dog breeds that were not involved in the mapping studies. We found that i) known *IGF1* and *HMGA2* loci variants for small body size are associated with separation anxiety, touch-sensitivity, owner directed aggression and dog rivalry; and ii) two loci, between *GNAT3* and *CD36* on chr18, and near *IGSF1* on chrX, are associated with several traits, including touch-sensitivity, non-social fear, and fear and aggression that are directed toward unfamiliar dogs and humans. All four genome loci are among the most highly evolutionarily-selected in dogs, and each of those was previously shown to be associated with morphological traits. We propose that the *IGF1* and *HMGA2* loci are candidates for identical variation being associated with both behavior and morphology. In contrast, we show that the *GNAT3-CD36* locus has distinct variants for behavior and morphology. The chrX region is a special case due to its extensive linkage disequilibrium (LD). Our evidence strongly suggests that sociability (which we propose is associated with *HS6ST2*) and fear/aggression are two distinct GWA loci within this LD block on chrX, but there is almost perfect LD between the peaks for fear/aggression and animal size.

**Conclusions:**

We have mapped many canine fear and aggression traits to single haplotypes at the *GNAT3-CD36* and *IGSF1* loci. *CD36* is widely expressed, but areas of the amygdala and hypothalamus are among the brain regions with highest enrichment; and *CD36*-knockout mice are known to have significantly increased anxiety and aggression. Both of the other genes have very high tissue-specificity and are very abundantly expressed in brain regions that comprise the core anatomy of fear and aggression – the amygdala to hypothalamic-pituitary-adrenal (HPA) axis. We propose that reduced-fear variants at these loci may have been involved in the domestication process.

**Electronic supplementary material:**

The online version of this article (doi:10.1186/s12864-016-2936-3) contains supplementary material, which is available to authorized users.

## Background

It is difficult to perform genomewide genetic association studies (GWAS) of human behavior. This is due to heterogeneity, biological complexity, ambiguous phenotype classifications and the challenges of phenotyping large numbers of individuals. As a result, very little is known about human behavioral genetics. Most of the progress has been driven by the availability of epidemiological data of medical relevance: smoking behavior [1], coffee consumption behavior [2], alcohol drinking behavior [3], and mental disability [4] or illness [5]. In contrast, there has been limited exploration of common complex-behaviors such as aggression [6], happiness [7] or social phobia [8].

Largely due to heterogeneity, most complex traits are difficult to map in humans. Dozens to thousands of variants can each contribute minute amounts to heritable risk, and this can differ dramatically in different ethnicities and their subgroups. Many major breakthroughs in human genetics have resulted from studying isolated populations and multigenerational families. The advantages of those latter approaches, and others, are dramatically exaggerated in dogs: (i) There are approximately 400 dog breeds, each on the order of 100-fold less genetically-complex than the full population. Thus, compared to humans and their major ethnic groups, dogs are much more similar within breeds and much more different across breeds. (ii) Dogs are often part of a family or working environment and receive high levels of health care. Lastly, iii) dogs have more phenotypic variation than any other land mammal; and much of that variation is the result of “evolutionary” selection under domestication. The strengths of dog models of complex genetics have been exploited mainly in the area of cancer [9], but recently also in behavior. Examples include investigation of obsessive compulsive disorders in select breeds [10, 11] and diverse behavioral traits in one breed (nerve stability, affability, wariness, adaptability, sharpness, activity and reactions during blood drawing [12]).

Strikingly, multiple groups recently showed that most genetic variation associated with diverse morphological traits in dogs can be mapped by cross-breed GWA using only breed stereotypes and about a dozen or more individuals each from dozens of diverse breeds [13–15]. This revealed that a great extent of the genetic variation in domesticated dogs was already present prior to breed creation. A good example is the trait of body size, the majority of which is explained by six gene variants in all but very large breeds (see the following and refs within: [16]). Those canine genes are relevant to known size-variation related genes across phyla (e.g., *IGF1*, IGF1 receptor, growth hormone receptor, *HMGA2* and *SMAD2*) and others indicate opportunities for discovery (e.g., *STC2*). A landmark GWAS from 2008 reported many loci and five candidate genes associated with the following behavioral traits: herding, boldness, excitability, pointing, and trainability [13]. A more recent study that used the same stereotype data, and a cohort with different breeds [15], did not replicate those findings for one overlapping trait, boldness (which was associated with loci on five chromosomes in the first study and a single other chromosome in the second). The chr10 region that was associated with boldness in the newer study, between the *MSRB3* and *HMGA2* genes, was the same as was strongly associated with two morphological traits – reduced ear erectness and small size. Although each of the three traits appeared to be associated with a different haplotype, with one exception, all bold breeds were erect-eared and small, and vice versa for non-bold breeds. This region spans among the most highly-differentiated markers reported from single-marker *F*_*ST*_ analysis and, at 2 Mb, it is the second largest of such regions. Similarly, Vaysse et al. showed that sociability (attitude toward unknown humans) maps to the highest *F*_*ST*_ region in the genome (2.6 Mb, chrX), which was shown by others to be associated with skull shape and large size [14]. To our knowledge, there are no further claims to resolve the various genetic associations or to suggest biological relevance of those loci to boldness and sociability; they appear to be open questions.

Here we report mapping fear and aggression traits associated with genetic variation shared across diverse breeds. These represent very common and important canine traits in the behavioral veterinary setting [17], and in human public health [18]. It seems likely to us that our findings will also prove to be relevant to human anxiety disorders and aggression, violence and criminality. Additionally, dog is the only animal that was originally domesticated by humans for almost-purely behavioral traits – and arguably is the only predator to be fully domesticated. Fear, aggression and related traits like tameness have long been thought to be central to the domestication of dogs [19], and this is supported by experimental domestication of silver foxes [20]. Both wild wolves and foxes are typically more fearful and aggressive than their domesticated counterparts; however, some dog breeds have been actively selected for enhanced aggressiveness in certain contexts such as fighting, guarding or vermin control. Our findings show that canine fear and aggression that are directed toward strange humans or other dogs share variation that was present prior to the creation of dog breeds. Fine mapping of those two loci implicates genes that are strongly suggestive of having relevance to fear/aggression. One variant is protective and the other increases risk of fear and aggression. We discuss below how variation at these loci may have been selected-for during the process of domestication.

## Results

### Study design

The present study was designed to test whether breed stereotypes of fear and aggression could be mapped by cross-breed GWA. While this concept has been validated for morphological traits, it has not been for behavioral traits. Success here requires two primary elements: biologically-relevant and robust phenotype data (seemingly likely from studies cited below), and the sharing of behaviorally-associated genetic variation across diverse breeds (which is unknown). We used three unrelated breed-specific resources: one of behavioral phenotypes [21] and two of breed-specific genotypes [14, 15]. The phenotype dataset is derived from C-BARQ owner questionnaires [22]. In C-BARQ, fear and aggression comprise five and four subtypes, respectively. All but two of these C-BARQ phenotypes (‘dog rivalry’ and ‘touch sensitivity’) were previously validated using a panel of 200 dogs with prior diagnoses of specific behavior problems [22]. More recently, other studies have also provided criterion validation by demonstrating associations between these phenotypes and particular training outcomes in working dogs [23], and the performance of dogs in various standardized behavioral tests [24–26]. There is currently no alternative phenotype resource that approximates the numbers of breeds and traits represented. The two genotype datasets used here are those used to map cross-breed stereotypes and study population genetics by Vaysse and Ratnakumar et al. (a large European collaboration including the LUPA Consortium, led by Webster; 30 breeds, 175,000 SNP markers) and Boyko et al. (a large USA collaboration led by Bustamante and Ostrander; 45,000 SNPs). The behavioral and genotype datasets overlap for 11 breeds and 29 breeds for Vaysse’s and Boyko’s datasets respectively.

Our study design included the following: i) principal components analysis (PCA) of breed phenotypes and genotypes (C-BARQ data and each genotype dataset); ii) discovery study of GWA mapping of published C-BARQ behavioral data (i.e., that corresponding to the Vaysse subset of the 30 most popular American Kennel Club breeds) with the Vaysse genotypes; iii) confirmation study of the Discovery results using C-BARQ and Boyko datasets; iv) testing of the internal-consistency of the C-BARQ phenotypes and of the prediction-model performance in a second set of breeds for which phenotype data was not previously published; and v) fine mapping and biological relevance analysis of the two peak regions associated with canine fear and aggression directed to other dogs and human strangers.

### Discovery studies of genetic association with fear and aggression

Assuming sufficient power, the greatest potential limitations of the present GWAS’s are latent variables, such as cryptic relatedness and batch effects, and population structure. We mitigated these in several ways, beginning by using many breeds instead of few. We used two genotype datasets that represented different genotyping platforms and cohorts with partially overlapping breed contents as discovery and confirmation datasets. Thus, each dataset has different batch effects, population structure and cryptic relatedness. Lastly, we controlled for relatedness and population structure in the GWAS’s by using a centered relatedness matrix correction (i.e., the Genome-wide Efficient Mixed Model Association algorithm or GEMMA [27]).

**Fig. 1:**
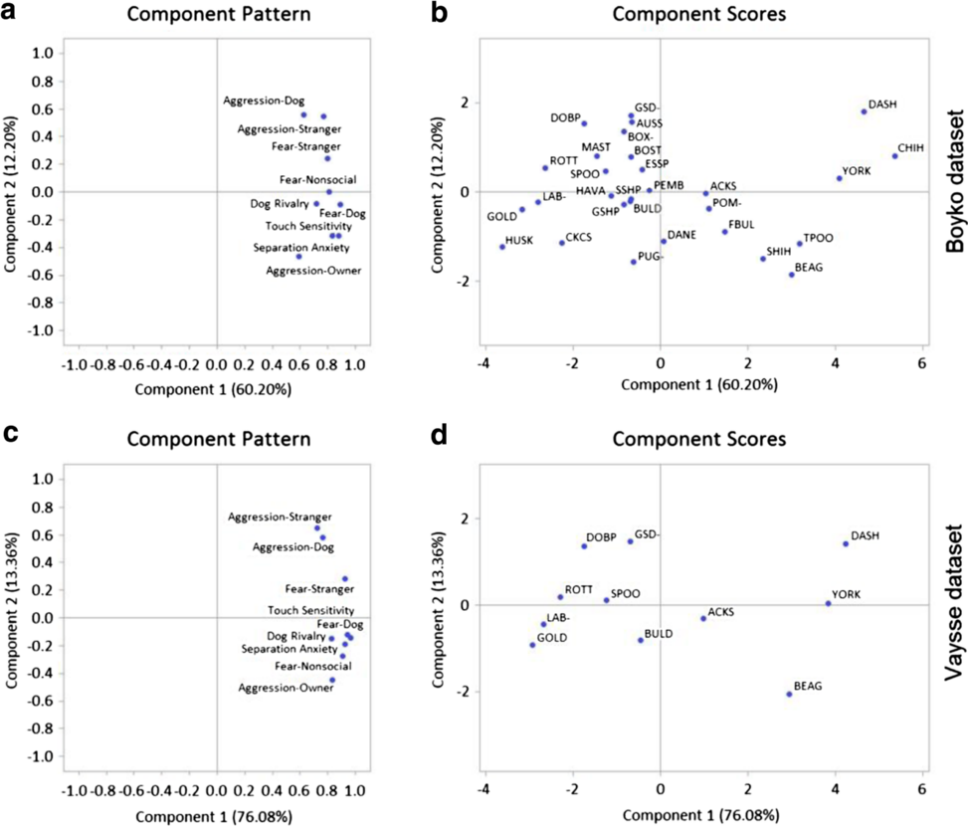
PCA analysis of behavioral traits. Since the two GWA datasets only partially overlapped in breed content, PCA analysis was performed to evaluate if the breed makeup affects the distribution structure of the variables. **a**, **b** Component pattern and component scores, respectively, of C-BARQ behavioral traits on the matching breeds in the Boyko dataset. **c**, **d** Component pattern and component scores of C-BARQ behavioral traits on the matching breeds in the Vaysse dataset

Before initiating mapping studies, we conducted Principal Components Analysis (PCA) of the breed-specific C-BARQ data on fear and aggression (Fig. 1). Component scores of the breeds evaluated (Fig. 1b/d) showed no relevant deviations across the two genetic datasets. Breeds evaluated in both datasets are distributed similarly in both plots. This preliminary evaluation suggests that, despite having different breed makeups in the two datasets, they show consistent results. Using either genotype dataset the following traits are clustered together apart from the others: stranger-oriented fear, stranger-directed aggression and dog-directed aggression. This suggested to us that the three traits could be genetically-related. A second implication of the PCA results is that owner-directed aggression is most distant, and therefore different, from the three clustered traits mentioned above. In other words, the PCA indicated a testable hypothesis that the three clustered traits would share associated loci, but that owner-directed aggression was associated with other loci (we go on to confirm this).

**Fig. 2:**
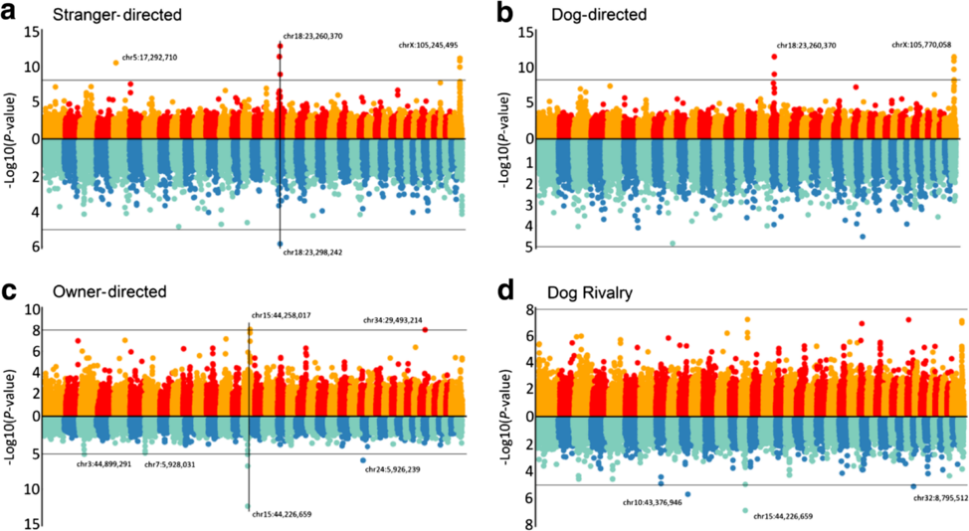
Manhattan plots of C-BARQ aggression traits. Vertical lines indicate relevant and consistent hits across the two GWA datasets. In each panel, the top plot corresponds to the Vaysse dataset while the bottom plot corresponds to Boyko dataset. **a** Stranger-directed aggression. **b** Dog-directed aggression. **c** Owner-directed aggression. **d** Dog rivalry

**Fig. 3:**
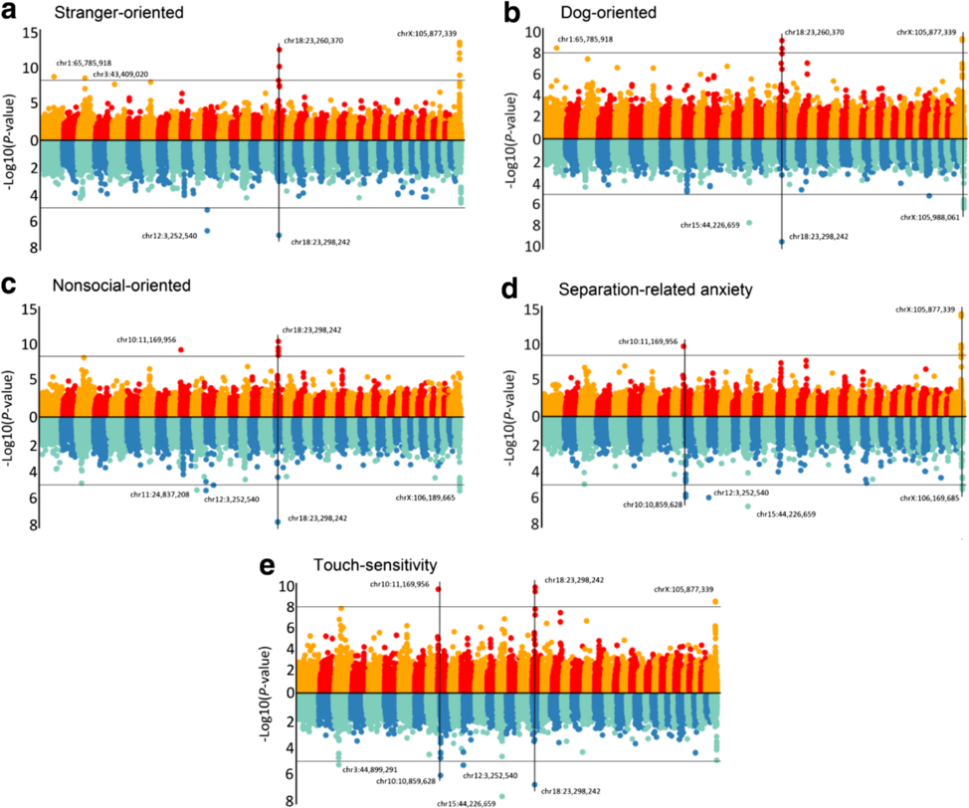
Manhattan plots of C-BARQ fear traits. Vertical lines indicate relevant and consistent hits across the two GWA datasets. In each panel, the top plot corresponds to Vaysse dataset while the bottom plot corresponds to Boyko dataset. **a** Stranger-oriented fear. **b** Dog-oriented fear. **c** Nonsocial-oriented fear. **d** Separation-related anxiety. **e** Touch-sensitivity

Our initial discovery study tested for genomewide-significant association for fear and aggression traits from C-BARQ using the genotype dataset of Vaysse et al. [15] (Figs. 2 and 3). This GWAS involved 175,000 SNP markers genotyped in 150 individuals from 11 breeds. Consistent with the PCA results, stranger-oriented fear, and stranger and dog-directed aggression is predominantly associated with many markers at two loci – chr18:23,260,370 and chrX:105,245,495-105,877,339 (CanFam2 assembly) – whereas owner-directed aggression is distinct. Rather, the latter is associated with previously described small-size variation in the *IGF1* gene on chr15:44 Mb and with a single marker at chr34:29 Mb (Fig. 2c). Dog-oriented fear, which does not purely cluster with stranger-directed fear/aggression and dog-directed aggression (but is not distant), is also predominantly-associated with the chr18 and X loci. Touch-sensitivity also has both of those loci, but a marker on chr10:11 Mb (affecting *HMGA2*; in this dataset, it is the second strongest cross-breed variant associated with small size after *IGF1* [15]) has the second strongest signal and chrX:105 Mb is considerably weaker. These latter two findings are consistent with the PCA pattern in Fig. 1c, where touch-sensitivity and dog-oriented fear are closest to stranger-oriented fear and stranger- and dog-directed aggression. The other fear traits share one, but not both, of the chr18 and X loci: nonsocial fear is associated with chr18 and the same chr10:11 Mb marker associated with touch-sensitivity; and separation-related anxiety is associated with chrX:105 Mb and chr10:11 Mb (chr18:23 Mb is suggestive).

### Confirmation of genetic association results in a second cohort

We repeated the discovery studies using the same behavioral data, but with the Boyko genotype dataset [14] (Figs. 2, 3; Table 1). The data we used was genotypes of 45,000 markers in 327 individuals from 29 breeds, 11 of which overlapped the Vaysse data used above. Because the same breeds were not used in the two studies, this is not strictly a replication study. However, because we are looking at cross-breed association, the findings in one can confirm those in the other. We also expect the paired GWAS’s to mitigate false positive hits that are due to latent variables or population structure in the individual studies.

**Table 1:**
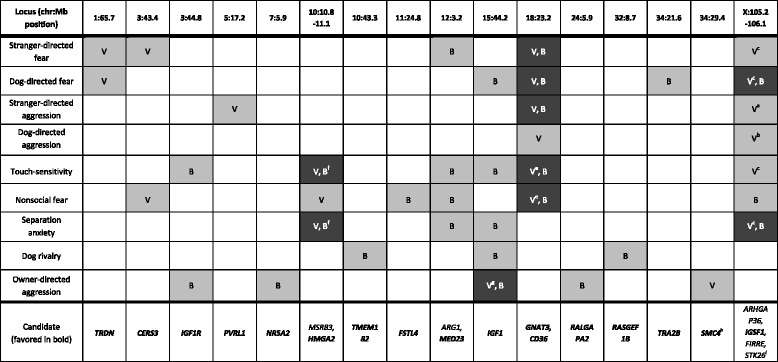
Summary of canine fear and aggression GWAS results. Fear and aggression trait peaks are given for separate GWAS studies using Vaysse (marked “V”; Illumina HD) and Boyko (“B”; Affymetrix v.2) genotype datasets. Loci shared with both are black and others are gray. Coordinates use CanFam2 assembly

^a-d^The peak SNPs chrX:105,245,495, chrX:105,770,058, chrX:105,877,339, and chrX:106,189,665 (numbered ^a-d^ in superscript, respectively) lie within one LD block. At least SNPs 2 and 3 are presumed to implicate the same haplotype/functional variant; candidate genes refer to these peaks

^e^The peak SNP is 23,298,242 (vs. chr18:23,260,370 for the others)

^f^The peak SNP for Vaysse is chr10:11,169,956 and for Boyko is chr10:10,859,628

^g^Vaysse peak SNP chr15:44,258,017; Boyko peak SNP chr15:44,226,659

^h^Peak SNP is a coding variant at a generally mammalian-conserved position

^i^ *ARHGAP36*, *IGSF1* and long non-coding RNA *FIRRE* are co-expressed, including in the pituitary gland and hypothalamus (see text)

The results of the confirmation GWAS’s generally confirmed associations of chr18 and X with stranger and dog-oriented fear and aggression. Both loci are genomewide-significant in dog-oriented fear, but only chr18 is significant in stranger-oriented fear and aggression (chrX was suggestive in both). Dog-directed aggression has no significant hits, but has suggestive evidence for chr18 among the top ten markers. As in the Vaysse GWA above, owner-directed aggression is most strongly associated with several markers that peak within the *IGF1* gene on chr15:44 Mb. Whereas the discovery GWAS of dog rivalry has no hits, the confirmation GWAS shows strong association with *IGF1*; and, unlike in the discovery GWAS, this locus is also significant in dog-oriented fear, separation-related anxiety, and touch-sensitivity. We interpret this as predominantly due to the breed make-up of the two cohorts. At the level of genomewide significance, the most similar results between the two studies are for dog-oriented fear (chr18 and X), separation-related anxiety (chr10 and chrX) and touch sensitivity (chr10 and chr18, with chrX being significant in discovery and almost significant in confirmation).

### Prediction model and internal consistency of C-BARQ behavioral data

**Fig. 4:**
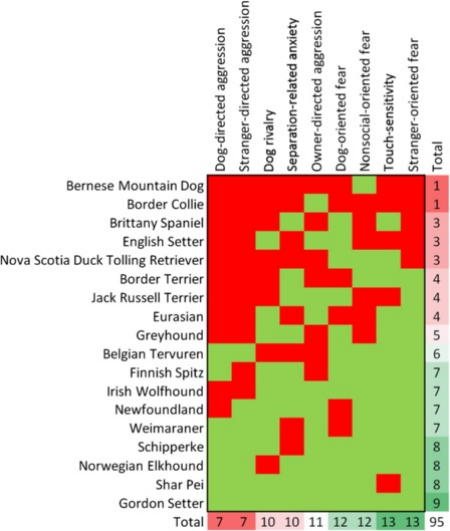
Success/failure matrix of predicted values. Green fill indicates a successful prediction and red is a failed prediction. Columns correspond to aggression and fear traits while rows correspond to dog breeds predicted. Totals are the sum of successful prediction within the column/row; columns and rows are sorted in numerical order and cells have a fill color gradient that goes from red (worse) to green (best)

We next created a model to predict fear and aggression behavior in a third group of dog breeds not included above. In the Additional file 1, we provide a detailed description of the methods and results. The goal was to test the predictive potential of the loci identified in the discovery and replication phases of this study. We thus tested a model of the discovery loci applied to a set of dogs breeds not included in the discovery/replication cohorts mentioned above. Briefly, we used the allele frequencies for the four main loci (only chr10:11169956, chr15:44258017, chr18:23260370, chr18:23298242 and chrX:105245495, chrX:105770058 and chrX:105877339 were used) to define a multiple linear regression by using a stepwise forward selection method (Additional file 2: Figure S1). Chr18 and chrX contributed significantly in the model for most traits whereas chr15 was only significant for owner directed aggression. We evaluated the performance of our predictive model and determined that it had a 57.9 % success rate, which is significantly higher (*P* < 0.0001) than the 6.7 % random chance of success (Fig. 4). We observed that some breeds are harder to predict which suggests that breed specific variants are likely to exist.

### Haplotype analysis and signatures of positive selection

**Fig. 5:**
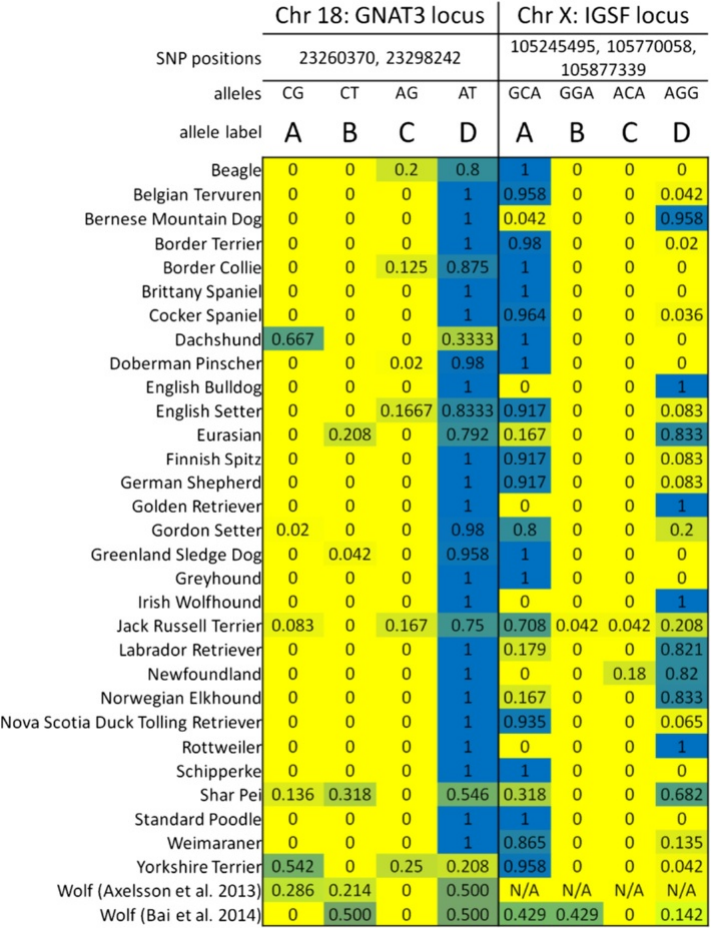
Haplotype distribution across dog breeds based on novel chr18 and X markers associated with aggression and fear. Haplotypes were defined only on the alleles of the top markers detected in this study. Allele distributions are color coded on a gradient that goes from yellow (fixed for allele 1) to blue (fixed for allele 2). Allele label letters are arbitrary

**Fig. 6:**
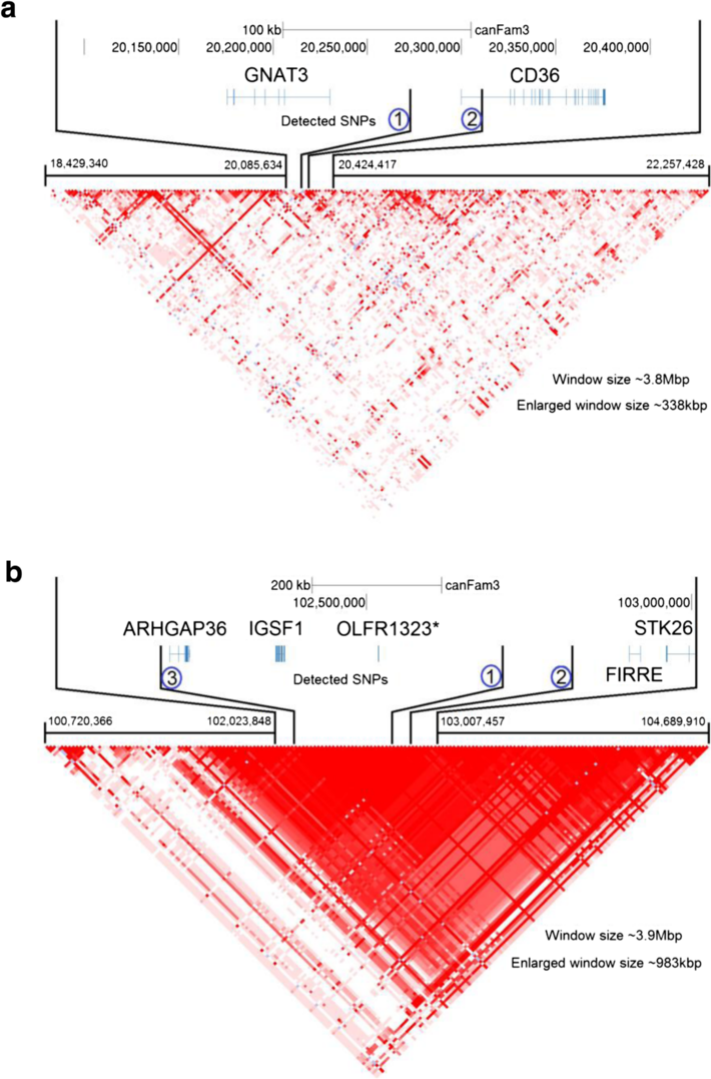
Linkage Disequilibrium plots for chr18/X fear and aggression loci. **a** Chr18 GWA locus for fear/aggression (using C-BARQ phenotypes and the Vaysse genotype dataset). **b** ChrX locus for the same GWAS as **a**. Genomic coordinates are converted from CanFam2 to CanFam3, and gene information is from the Broad Institute’s CanFam3 Improved Annotation Data v.1. * OLFR1323 is a mouse name gene; in dog, the name is ENSCAFG00000018811 (Protein Coding Gene). Linkage disequilibrium plot was created with Haploview v.4.2

The associated loci and alleles on chr15 (*IGF1*), chr10 (*HMGA2*) and chr3 (*IGF1R*) are the same as those known to be associated with small size across dog breeds (reviewed in [16]), including in the genotyped cohorts used here [14, 15]. Allele frequency (Fig. 5) and haplotype analysis of the peak region on chr18 reveals a very low level of LD (Fig. 6a), indicating that the associated haplotype is very old. In contrast to chr18, the associated region on chrX lies within a 2.6–3.7 Mb block of strong LD (Figs. 5 and 6b; [14, 15]). Analysis of relevant breeds using whole genome sequence data [28, 29] and DNA copy number variation (CNV) data [30–32] appears to rule out that the functional variants are protein coding changes or CNVs.

The peak GWAS hits on chr18 and X were further analyzed for signatures of positive selection. In addition to the fixation index (*F*_*ST*_*;* when several breeds share founder variation), we refer to statistics of population differentiation (*D*_*i*_; designed to detect selection in one or few breeds out of a larger group; pairwise *F*_*ST*_ values are normalized for a breed vs. the genomewide average, then summed across pairwise combinations involving that breed [33]) and reduced heterozygosity (*S*_*i*_; the sum of regional deviations in levels of genomewide relative-heterozygosity between two breeds is compared to the genomewide average, and the sum of those across all pairwise comparisons is calculated [15]). Genetic hitchhiking generally refers to neutral variation that is carried along with an allele under positive selection [34]. As the selected variant goes to fixation, there is a loss of variation flanking that site (termed a selective sweep) [35, 36]. LD in such a selected region increases dramatically, and that effect is the basis of several approaches for identifying positive selection (e.g., extended haplotype homozygosity, which detects large haplotypes suggestive of selective sweeps [37]). We searched for evidence of hitchhiking by measuring haplotype sizes through direct phasing analysis (see Methods).

**Fig. 7:**
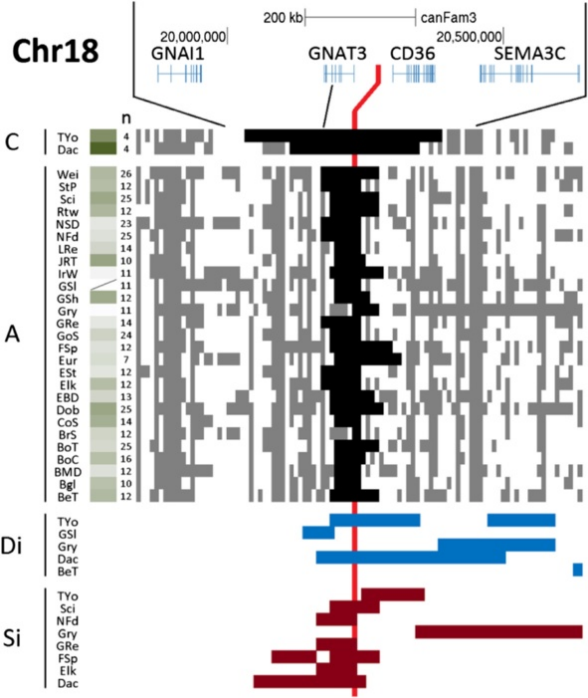
Mapping phased-haplotypes and *S*_*i*_/*D*_*i*_ regions for fear/aggression traits. Quantitative trait with increased-fear/aggression associated with the C allele. Letters on the left indicate the fixed allele required for phasing. Red line is the peak SNP for fear/aggression GWA. The large window size corresponds to 1.50 Mb, and small to 812.1 kb. Green-white gradient shading represents larger-smaller fear/aggression risk. Diagonal lines indicate missing phenotype data

In Fig. 7 we present analysis of the new fear and aggression locus on chr18. Haplotype phasing analysis shows the two breeds with the increased risk allele (chr18: 23,260,370 and chr18:23,298,242) – Dachshund and Yorkshire Terrier – have much larger haplotypes compared to the alternative allele (ranging up to 683 vs. 186; minimal overlap regions of 418 vs. 13.2 kb). Their haplotype sizes, and the central position of the putative variant under selection, suggest a recent selective sweep according to hitchhiking theory. Notably, only a subset of the increased-fear/aggression haplotype under selection contains the *FGF4* retrogene insertion that causes chondrodysplasia in some breeds but not in others. All known exceptions to the co-occurrence of the retrogene insertion and the phenotype are very small dogs – Yorkshire Terrier, Chihuahua and Japanese Chin – leading those authors to propose the trait is not manifest in carriers that are small [38]. [That study included four Yorkshire terriers that had an insertion-allele frequency of 50 %; in our phasing analysis of this breed, we found 4 dogs homozygous for the increased-fear allele (Fig. 7) and 3 homozygous for the alternative allele (which reveals a short phased haplotype spanning 8 SNPs)]. Moreover, although some breeds have the retrogene insertion within the ancestral increased-fear/aggression haplotype on chr18, several breeds have >20 % allele frequency of that haplotype but lack the insertion and chondrodysplasia [38]. The following are those breeds that commonly carry the ancestral haplotype and are known to lack the retrogene insertion at a common frequency (in parentheses, the numbers of subjects genotyped): Beagle (*n* = 8); Border Collie (*n* = 4), Cocker Spaniel (*n* = 8), English Setter (*n* = 2) and Jack Russell Terrier (*n* = 7). Although the numbers of dogs screened for two of those breeds are small, none of the breeds has been reported to have heritable chondrodysplasia – which is dominant for this chr18 variant. Shar-Peis and Huskies, which are also common carriers of the ancestral haplotype, have not been screened for the retrogene insertion but have not been reported to have heritable chondrodysplasia.

Thus, there is evidence of recent selection for the chr18 increased-fear/aggression haplotype in Dachshunds and Yorkshire Terriers (large haplotype size); that selection is presumed to be for short legs in the former but unknown for the latter. Only the two breeds that carry the chr18 increased-fear/aggression allele at high frequency have *D*_*i*_ signal spanning the peak SNP, and both also have the longest *S*_*i*_ signal either intersecting or close to the peak SNP. The allele protective of fear and aggression has high allele frequencies in all but the two breeds that carry the risk allele, and is fixed (>95 %) in 22 of the 30 breeds (5 others are ≥75 %). In contrast, two sources of wolf sequence data from around the world show the wolf allele frequency of the two alleles to be ~50 %. We propose these patterns are consistent with selection during the domestication process. If so, that selected reduced-fear and aggression variant is narrowed to a two-SNP minimal overlap interval of phased haplotypes.

**Fig. 8:**
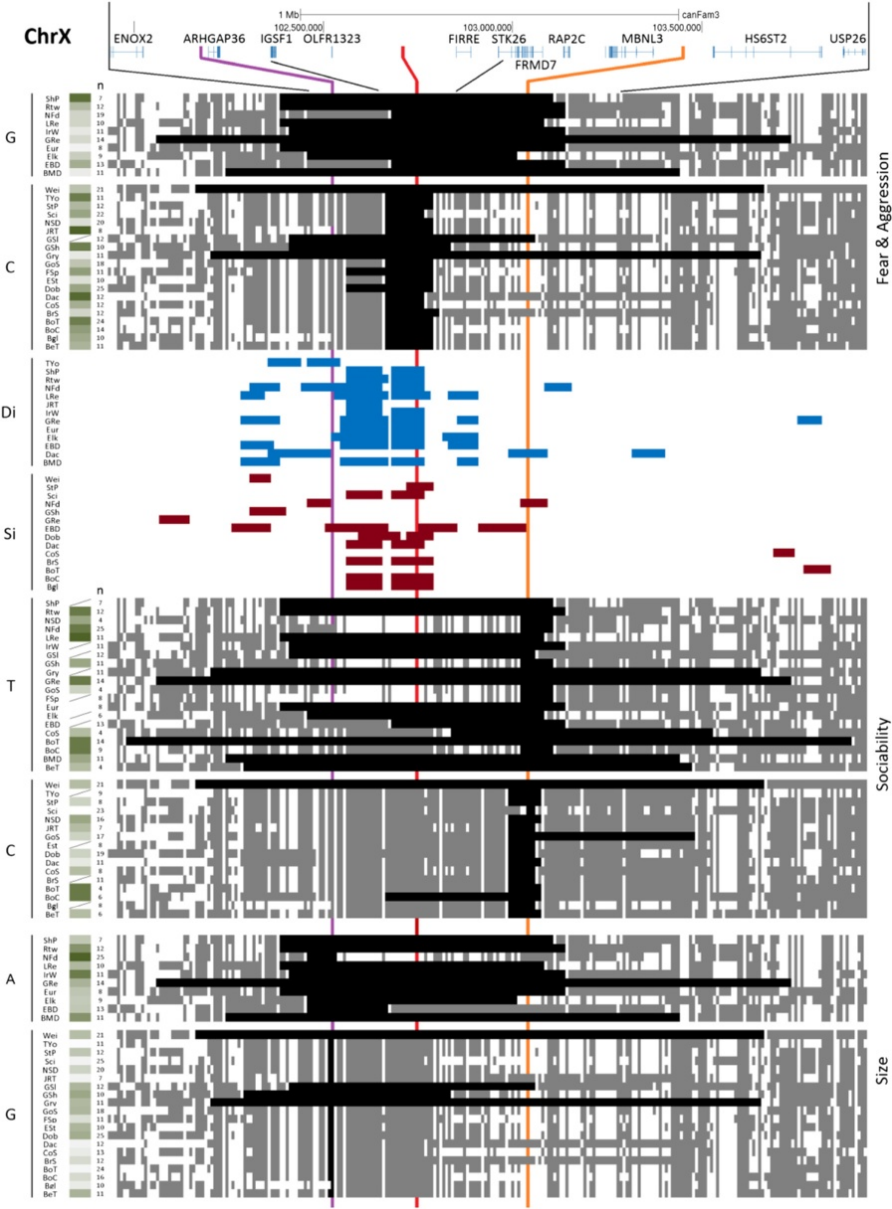
Mapping phased-haplotypes and *S*_*i*_ /*D*_*i*_ regions for fear/aggression traits. Quantitative trait with decreased-fear/aggression associated with the G allele. Letters on the left indicate the fixed allele required for phasing. Red line is the peak SNP for fear/aggression GWA, orange is for sociability peak and purple is for size peak. The large window size corresponds to 5.20 Mb, and small to 2.00 Mb. Green-white gradient shading represents larger-smaller fear/aggression risk. Diagonal lines indicate missing phenotype data. * OLFR1323 is a mouse name gene; in dog, the name is ENSCAFG00000018811 (Protein Coding Gene)

In Fig. 8 we present a 5 Mb chrX region that contains GWA peaks for size, fear and aggression, and sociability. As described above, this region has the highest *F*_*ST*_ level in the dog genome and has strong LD across 2.6–3.7 Mb [14, 15]. The overall pattern of phased haplotypes and *D*_*i*_ and *S*_*i*_ signal suggests the three traits may be distinct. The fear/aggression peak overlaps *D*_*i*_ and *S*_*i*_ signal for many breeds, but that for size and sociability do not. However, there is perfect LD between the size and fear/aggression peaks in this haplotype in 9 of 11 breeds, and more data is necessary to establish their relationship. Human mutations in the top chrX candidate gene for fear/aggression, *IGSF1*, are known to affect human growth hormone biology [39], suggesting the two dog traits could share all or some genetic variation at this locus; additionally, expression of the gene abutting the peak SNP for size, *ARGHAP36*, is strongly correlated with *IGSF1* expression (see below). Although all but one of the breeds with the fear/aggression-protective allele have perfect LD with the sociability allele, only half of the breeds with the sociability allele have the fear/aggression-protective allele in perfect LD. All breeds with the reduced-fear/aggression allele show *D*_*i*_ signal overlapping the fear/aggression peak, but there is only one segment of each overlapping the sociability peak. On the other hand, there are eight *S*_*i*_ segments at the fear and aggression peak (one is shifted a single SNP away), but none is in a breed with the fear/aggression-protective allele.

The predominant signatures of positive selection on the chrX locus thus point to the association with fear and aggression. This is also suggested by the phased haplotype analysis, which shows minimal overlapping regions of 841 vs. 284 kb for the fear/aggression-protective and alternative alleles, respectively. In contrast, the appearance of such an effect at the sociability locus is due to the contribution of the 10 breeds that carry the fear/aggression-protective allele in perfect LD; and the minimal overlapping regions there are 85 vs. 47 kb for the social allele and the non-social allele, respectively. Notably, this locus presents the possibility of a special case of hitchhiking in which selection initially occurs on one functional variant (here reduced-fear/aggression), but a second variant in the region is also favored in subsequent selection (here, size or sociability). As a result, relative to the original variant selected, the LD breaks down only on the side opposite the second selected variant.

Based on modern worldwide wolf sequences, the minor chrX allele is that associated with increased-fear/aggression (43 % frequency) [29]. Of the diverse 30 breeds studied here, 20 have frequencies >70 % for the increased-fear/aggression allele and 10 have such frequencies for the alternative allele (with many breeds fixed for one of those). In the discussion we present the considerations for interpreting this.

### Virtual fine-mapping of fear and aggression loci on chr18 and X

We next interpreted the peak GWAS signals and signatures of selection to call the minimal intervals for fear and aggression on chr18 and X. Vaysse et al. showed that top ranked *S*_*i*_ and *D*_*i*_ signals mirror each other in a subset of regions, but the effects are highly variable; and interpretation seems complex and lacking fine resolution. Our own experience suggests that *D*_*i*_ signal is generally more sensitive and precise for fine mapping, but many regions appear to be flagged by reduced and more-diffuse signal of both statistics. Here we propose the following scheme: i) the maximum map interval corresponds to the region of overlap of phased haplotypes across all breeds (separately applied to both SNP alleles at a GWA peak); and ii) the minimum map interval can combine that with the smallest region of overlap of *D*_*i*_ or *S*_*i*_ regions across breeds. In accordance with hitchhiking theory, it seems likely that the maximum map interval for a region under selection will always capture the functional variant. However, it is not clear how precisely that critical region could be narrowed using different signatures of selection; each case could be unique (e.g., due to soft vs. hard sweeps) and is likely to require additional confirmation.

For the chr18 fear and aggression locus, the predicted maximum map interval for the most recently selected increased-fear/aggression haplotype is 418 kb and the minimum is 108 kb. However, if there was initially selection for the reduced-fear/aggression allele at domestication and subsequent selection for the increased-fear/aggression allele at the same position (as we speculate based on parsimony), then the maximum and minimum map intervals would be 13.2 kb. For the chrX locus, the predicted maximum map interval for the reduced-fear/aggression allele is 841 kb, and the minimum map interval is 218 kb. The sociability locus lacks significant *D*_*i*_/*S*_*i*_ evidence and has no phenotype for some of the breeds segregated for the fear and aggression phenotype. The selected sociability allele T thus has maximum and minimum map intervals of 147 kb. Notably the original report of this sociability GWA, using the same genotype data, was only suggestive in the full dataset; rather, genomewide significance was detected in analysis of females exclusively, and yielded a peak of 10 SNPs spanning 580 kb [15].

### Biological relevance of candidate genes

According to the minimal intervals, we have implicated the following genes: i) in the chr18 fear and aggression locus, *GNAT3* and *CD36*; ii) in the chrX fear and aggression locus, *IGSF1*, *FIRRE* (long non coding RNA) and STK26; and iii) in the chrX sociability locus, *MBNL3* and *HS6ST2*. Both sociability candidates are expressed in the brain: *MBNL3* at very low levels and apparently not at all enriched in brain, but *HS6ST2* at very high levels and highly enriched in many brain regions (BioGPS microarray data for 176 human and 191 mouse tissues and cells [40]; NCBI GEO GSE1133 and GSE10246). We favor *HS6ST2* for the sociability association. For the fear and aggression loci, we only see clear biological relevance (mainly related phenotypes in mutant mice or expression in sensory organs, brain or adrenal gland) for *GNAT3, CD36* and *IGSF1*.

*GNAT3* encodes Gustducin alpha, the G alpha subunit that transduces taste receptor signaling. Gustducin alpha also has chemosensory roles in the vomeronasal organ, airways and gastrointestinal tract [41, 42]. There are also reports that *GNAT3* is expressed in areas of the brain that include the brainstem, hypothalamus and hippocampus [43–45]. However, those studies targeted specific brain regions and we are not aware of systematic analysis of the entire brain in any mammal. We consulted the Allen Brain Atlas of *in situ* mRNA hybridization analysis [46] and found evidence that *GNAT3* is most highly expressed in the amygdala in the adult mouse, specifically in layer 2 of the Cortical Amigdalar Area (Additional file 2: Figure S2). That finding is supported by analysis of public gene expression data showing that the highest ranked expression-change of *Gnat3* mRNA in any brain region is a 3.73-fold increase in the amygdala of rats 6 h after pain exposure (Nextbio analysis server [47]: *Gnat3* has a percentile gene-ranking score = 99 for the microarray experiment, p = 0.0165; NCBI GEO Accession GSE1779 [48]). The next-highest ranked (and significant after multiple testing correction) gene expression-changes reported in experimental paradigms involving the brain follow: i) rat hippocampus (GSE3531, score = 82); ii) mouse cortex (GSE31840, score = 81); and iii) mouse striatum (GSE48955, score = 65). In addition to very high expression in the amygdala, the Allen Brain Atlas of the adult mouse reveals lower levels of expression in parts of the pons: Lateral reticular nucleus, Paragigantocellular reticular nucleus, lateral part, and the Facial motor nucleus.

CD36 is an enigmatic protein known to be widely expressed and to have varied biological roles [49]. Its functions include chemosensory sensing and signaling (including in taste, pheromone and airway sensing), sensing and transport of fatty acids in diverse metabolic roles, high affinity binding and signaling of several molecules (including collagen and thrombospondin), and it is a subunit of cell surface scavenger receptors involved in phagocytosis. In the brain, the highest levels of *CD36* mRNA have been mapped to the Cortex-amygdala transition zone, Medial amygadaloid nucleus, posterior part, hypothalamic Premammillary nucleus, ventral part and and ependymal cells of the Central canal and Fourth ventricle; and high levels are also present in the Piriform cortex, Perirhinal cortex, Field CA1 of hippocampus, ventral, pyramidal cell layer, Basolateral amygdaloid nucleus, posterior part, Amygdalopiriform transition area, and Paraventricular thalamic nucleus, anterior [49]. Recent studies showed that *CD36*^-/-^ mice have behavioral phenotypes that include increased anxiety, aggression and locomotor activity [50].

*IGSF1* is Immunoglobulin superfamily member 1. Common human variation in *IGSF1* is associated with age at menarche, and loss-of function mutations cause a human syndrome of congenital hypothyroidism, macroorchidism, Prolactin and Growth Hormone deficiency, delayed pubertal testosterone and obesity [39, 51]. It is expressed very highly in the anterior pituitary and hypothalamus, but also in the choroid plexus, adrenal gland, pancreas, heart and skeletal muscle, fetal liver and testis [52]. To generate quantitative data, we used the BioGPS GeneAtlas of genomewide gene expression in 191 mouse tissues and cells. The increased-expression levels, relative to the median for all tissues are as follows: pituitary, 1,060-fold; hypothalamus, 340; amygdala, 26; heart, 22; spinal cord, 11; hippocampus, 10; placenta, 10; and nucleus accumbens, 5.5 (data is for probe 1433652_at). Immunohistochemical evidence clearly shows that IGSF1 protein expression is restricted to neurons (including at low levels in the cortex, lateral ventricle, and cerebellum [53]). Notably, there is strong evidence that *IGSF1* is co-expressed with its two flanking genes, *ARHGAP36* and the long non-coding RNA *FIRRE.* In the mouse BioGPS GeneAtlas of 191-tissues/cells, expression of *IGSF1* (probe 1433652_at) has Pearson correlations of 0.85 with *FIRRE* (1436638_at; two other *FIRRE* probes also have correlations >0.76) and 0.80 with *ARHGAP36* (1454660_at); the same probe has a correlation 0.92 with a second *IGSF1* probe. The human BioGPS GeneAtlas of 176 tissues/cells lacks a probe for *FIRRE*, but *IGSF1* and *ARHGAP36* have a correlation of 0.97 (probes 207695_s_at and gnf1h04904_at). It is thus possible that variation in the *IGSF1* region could also affect the other two genes.

## Discussion

Understanding fear and aggression in dogs is important for canine wellbeing, human public health and to understand the process of dog domestication. It also has great potential to lead to medical translation to related mental disorders in humans. Here we mapped behavioral traits by using breed stereotypes to conduct a series of interbreed genome scans. This approach is well developed for morphological traits, but has not been validated for behaviors. In this work, we validate our findings by conducting separate GWAS’s using cohorts comprised of partially overlapping breeds. We then provide additional validation by developing a predictive model and applying it successfully to a different group of breeds. This also serves to demonstrate internal consistency of C-BARQ phenotyping across breeds.

Our PCA of breed genotypes and phenotypes indicates that some types of fear and aggression are related to each other (stranger-oriented fear and dog-/stranger-directed aggression), but are distinct from others such as owner-directed aggression. This pattern was mirrored by the results of our behavioral GWAS’s which identified two genome loci associated with the former (also shared with dog-oriented fear) and another two associated with the latter (also shared with dog rivalry). Notably, owner-directed aggression and dog rivalry are associated with the same variation in *IGF1* that is known to have the greatest contribution to small-size across dog breeds (the former trait is also associated with the small-size variant at the IGF1 receptor gene). This finding is consistent with previous reports that i) there is a highly-significant correlation between the behaviors of owner-directed aggression and dog rivalry, ii) this correlation is independent of dog- and stranger-directed aggression, and iii) these behaviors are associated with breeds of small to medium size [54–56]. As owner personality does not necessarily predispose to owner-directed aggression, it is thus an apparent dog trait [57]. Some of those studies also showed a correlation between small size and stranger-oriented fear and aggression, dog-oriented fear, separation anxiety, and touch sensitivity [56]. That is supported by our finding in the confirmation GWAS’s that the same small-dog *IGF1* allele is associated with the latter three traits. It is unclear whether the behavioral associations with small-size gene variants are due to developmental, physiological or psychological effects; all seem probable.

The loci on chr18 and X are particularly interesting because they may have been originally selected for fear/aggression traits. The four principal loci discussed here – on chr10, 15, 18 and X – have experienced very strong selection (according to *F*_*ST*_ [14, 15]) and are associated with both behavior and morphology traits. For chr10 and 15, the evidence is consistent with the same or overlapping variation causing both traits at each locus. For chr18, the variants for behavior (increased fear/aggression) and morphology (short legs) are distinct. Some breeds, such as the Dachshund, contain the short legs mutation and the nearby increased-fear/aggression variation in the same haplotype (in some very small dogs that carry the short legs variant, such as the Yorkshire Terrier, that trait is not manifest in carriers of that chr18 chondrodysplasia mutation) [38]. Other breeds, such as the Beagle, Border Collie, Cocker Spaniel, English Setter and Jack Russell Terrier are common carriers of the same ancestral increased-fear/aggression haplotype on chr18, but are known to lack the chondrodysplasia mutation [38]. The chrX locus is a special case because it lies in a region of very strong LD. As a result, the question of independent trait-variants is moot because the original haplotype (that still persists very commonly) was perfectly-associated with size, skull morphology, reduced-fear/aggression and increased sociability. We are not aware that the unspecified skull morphology trait [14] is readily apparent, beneficial or the subject of human interest in any respect (vs. morphological traits selected in purposed breeds: e.g., short legs for chasing burrowing-animals or skull/jaw geometry and bone-thickness ideal for bull baiting). However, whether any of the chr18 traits was the primary focus of human selection, all of them would have been essentially-inseparable for a very long time after the initiation of domestication. The question of whether the chrX variation is specific for behavior or shared with that for animal size could be answered by identification and phenotyping of dogs that are recombinant between the two peaks; or, it could be settled by making caninized mice for the two alternative sequences at each peak region.

The closest genes to the chr18 and X association peaks are *GNAT3* (Gustducin alpha, the G protein alpha subunit for bitter, sweet and umami taste cells [58]) and *CD36*, and *IGSF1* (which results in a congenital syndrome affecting thyroid and growth hormones when mutant in humans [39]), respectively. The fear/aggression peak near *IGSF1* lies within a 2.6–3.7 Mb region of strong LD that includes flanking peaks for size and sociability [14, 15]. The reduced-fear/aggression allele on chrX is often in perfect LD with the increased-size allele, but much less so with regards to the increased-sociability peak allele. We suggest that the fear/aggression functional variants on chr18 and X are most likely to affect expression of *GNAT3* and/or *CD36*, and *IGSF1*, respectively. [Notably, *GNAT3* and *CD36* are co-expressed in taste cells and could also be in brain regions such as the amygdala and hypothalamus (see Results); and *IGSF1* is co-expressed with its two flanking genes, *ARHGAP36* and the long non-coding RNA *FIRRE* [40]]. Both of those neuronal genes have strong biological relevance at the level of neuroanatomy. Outside taste receptor and other chemosensory cells (and a subset of vomeronasal interneurons), *GNAT3* is most highly expressed in the amygdala. CD36 is most highly expressed in the Cortex-amygdala transition zone, regions of the amygdala and hypothalamus and ependymal cells of the Central canal and Fourth ventricle. *IGSF1* is predominantly and very abundantly expressed in two brain regions - the pituitary and parts of the hypothalamus [46]. Further studies are necessary to determine if the size and fear/aggression traits on chrX are due to the same, distinct or overlapping variation. It is interesting that at least three genes in the region are co-expressed in tissues involved in determination of body size (the pituitary gland and hypothalamus), and that mutations in *IGSF1* affect human size [39]. Importantly, new studies show that *CD36*-knockout mice have behavioral traits that include increased aggression, anxiety and locomotor activity [50]. Rat studies have revealed that mRNA expression of both other genes are regulated in fear-relevant models: *GNAT3* in amygdala under pain stimuli [48]; and *IGSF1* in cortex after stress or tactile stimuli [59]. Thus both genes are also strong candidates for fear relevance: they are neuronal and associated with stress/anxiety according to interbreed dog GWA, neuroanatomy and biology.

Because our model of the chr18 and X variation was successful in predicting the relevant fear and aggression behaviors in a third group of non-overlapping breeds, we believe these markers can be used to predict and, in part, explain such behavior across many dog breeds. However, the behavioral stereotypes of some breeds were not explained by our predictive model, and many breeds have not been tested. It seems likely that many breeds have epistatic variation with chr18 and X variants or have other variants that are less commonly associated with these traits across dog breeds. All of these issues can be addressed by studies of individual breeds. Since we have only found the common co-occurrence of the increased-fear/aggression variation on chr18 and X in small and medium dogs, it will be interesting to see if this is also present in large dogs bred for aggression or fighting.

Dogs were the first animals to be domesticated by humans. New studies indicate that dog domestication has a single origin in southern East Asia ~33,000 years ago, followed by migration to the Middle East, Africa and Europe ~15,000 years ago [60]. Canine fear and aggression are of great interest because those traits – mainly the loss of fear of humans – are widely believed to underlie the mechanism for domestication [19]. We thus considered whether the variation we identified at chr18 and X could have been involved in the process of dog domestication. Our analysis of published genetic variation from extant wolf populations across the world shows that both fear/aggression alleles (protective on chr18 and risk on chrX) are common in wolves – approximately 50 % [28, 29] and 43 % [29], respectively. In domesticated dogs, 27 of 30 breeds in Fig. 4 have the reduced fear/aggression chr18 allele at a frequency of at least 75 % (19/27 are fixed at a level of 100 %). In contrast, only 10/27 breeds have the reduced-fear/aggression chrX allele at frequencies greater than that of wolves (starting at 68 % allele frequency; 5 are fixed at a level of at least 95 %). The common high-frequencies of the chr18 reduced-fear/aggression allele across dog breeds are consistent with selection of reduced fear and aggression in the domestication of dogs. Interpretation of the chrX region seems less clear because the majority of breeds have the increased-fear/aggression allele. However, we show that the reduced fear/aggression allele has been under selection (i.e., its haplotype size is always large whereas the alternative allele is generally far smaller). The high frequency of the four alleles at chr18/X in extant wolf populations may seem counterintuitive in a model where ancestral wolves were generally more fearful and aggressive than domesticated dogs, but this could be explained by balancing selection or recent positive selection.

A concrete understanding of dog domestication may soon emerge, as there is a major effort underway to sequence ancient dog genomes [61]. Our prediction is that positive selection of the reduced-fear variants on chr18 (specific for this trait) and chrX (in strong LD with variants for large size and increased sociability) was part of the domestication process. This raises a previously unappreciated possibility – that the putative selection of less fearful/aggressive proto-domesticated dogs was of animals that were at the large end of the spectrum (which could introduce other relevant issues related to human selection and canine psychology and social behavior). For example, domestication of the largest wolves may have been favored for protection (incl. from wolves) or hunting large game (i.e., since they lacked the benefit of a true wolf pack). We further propose that humans have obscured those roots in modern dog breeds by selecting for increased aggression or for other linked morphological or behavioral traits (e.g., for short legs at chr18 or for increased aggression or smaller size at chrX). We expect there are many other fear/aggression-associated loci that are more difficult to map across breeds. But it seems likely that having two very common alleles influencing these behaviors led to frequent selection between those to set a level of reactivity and disposition towards dogs and humans. In this regard, it seems plausible that the correlation between small size and the highest levels of fear/aggression is because the same behavior in large dogs is generally unacceptable to humans [54]. In our principal mapping study, only small dogs – mainly Dachshund and Yorkshire Terrier – have high frequency of fear/aggression associated alleles at both chr18 and X. The fact that dog and stranger oriented fear and aggression are generally much more strongly associated with chr18 and X variants than with *IGF1*/chr15, *HMGA2*/chr10 and *IGF1R*/chr3 small-size variants further establishes that small size is not the predominant cause. However, it is also notable that a subset of large breeds carries the chrX reduced-fear/aggression and increased-size variants in perfect LD.

The biochemistry and neuroanatomy of the emotions of fear and aggression are highly conserved in vertebrates, and some argue this is true across the animal kingdom [62]. Across vertebrates the most immediate response to extreme threat involves the transmission of different sensory signals through the following sequence of brain regions (referred to as the low road): thalamus, amydgala, hypothalamus and pituitary gland, which sends nerve and hormonal signals to the adrenal glands, which in turn direct acute (through noradrenaline/adrenaline) and sustained (through glucocorticoid hormones such as cortisol) stress responses. This low road corresponds to innately programmed responses and is associated with emotions such as fear and anger. A parallel cognitive pathway that is referred to as the high road diverges at the thalamus, by then going to primary sensory and association centers in the cortex before continuing to the amygdala. Both pathways also involve bidirectional signaling with the hippocampus. Thus, while the immediate response to fear may be predominantly innate and emotional, it is not completely separate from cognition. There is extensive molecular and behavioral evidence that the hypothalamic-pituitary-adrenal (HPA) axis is the most critical driver of behavioral stress. Biochemical pathways implicated in social fear and aggression include signaling by serotonin and dopamine, and neuropeptides such as the predominantly-hypothalamic oxytocin and vasopressin [62]. Notably, the domestication of another canid, the fox, resulted in foxes with greatly reduced HPA activity [20]. After 45 generations of selection for tameness in foxes, basal blood cortisol levels were reduced three-fold and stress-induced levels five-fold (compared to normal foxes). Domesticated foxes also have increased levels of brain serotonin, consistent with its inhibitory effect on aggression. New analysis of selective sweep regions associated with domestication of pigs showed that *GNAT3-CD36* lies in one of the sweep regions of European (but not Asian) pigs [63]; but it remains to be seen if *GNAT3-CD36* variation is directly associated with that domestication event. Our findings that loci spanning *GNAT3-CD36* (which are highly expressed in the amygdala and hypothalamus) and *IGSF1* (highly expressed in the hypothalamus and pituitary gland) are associated with canine fear and aggression are thus consistent with a very large body of work implicating the HPA axis.

It is clear from animal and human studies that fear and aggression are often associated, but it is not always in the same direction [64]. Based on human behavior and pharmacology, the links between anxiety and aggression are very complex. Similarly, early life stresses in people and animals are associated in complex ways with anxiety disorders and aggression in adulthood. Early life stress generally involves changes in the HPA axis, and results in increased anxiety and altered social and aggressive behaviors. Animal models with a profile similar to the dog case presented here – in which both fear and aggression are elevated – are rare. Examples of knockout models that have this property include those for enkephalin [65] and α-calcium-calmodulin kinase II [66]. Selective breeding has also yielded strains that have increases in both anxiety and aggression. One of those is the North Carolina mouse, in which acute diazepam treatment reduces both anxiety and aggression [67]. The other example is a strain of Novosibirsk Norway rats that was bred for increased aggression to humans [68]. It is not immediately clear which human conditions may be most relevant to the present dog model at chr18 and X. Most likely those will include anxiety disorders. Notably, some anxiety conditions are associated with increased aggression, and this includes a subset of those affected by social anxiety.

## Conclusions

We have identified common variants associated with fear and aggression across dog breeds. These can now be biologically dissected at the levels of development, epigenetics, neuroanatomy, physiology and behavior (most powerfully in breeds segregating both alleles at any of these loci) or in genetically-modified rodent models. Among the areas of research on fear and aggression [62] that are ongoing in dogs and will be vastly accelerated by genetic handles are i) development and environmental-malleability [26, 69]; ii) molecular/biochemical and imaging descriptions at baseline and under acute stress [20], iii) effects on mental and physiological states in the life course [23–25, 54], and iv) feasibility to mitigate negative effects through cognitive or pharmacological treatments [70, 71]. In parallel, it will be important to determine the molecular mechanisms of these fear/aggression variants, and to identify their interactions with other genes and environmental factors.

## Methods

### Experimental design overview

Since the analysis of dog breed data is highly vulnerable to population structure issues and false positive detection, we designed our analysis in two phases: first, a discovery phase, where available SNP data was used to map aggression and fear behavioral traits. We designed this phase to analyze two independent SNP datasets using nine independent behavioral phenotypes for aggression and fear. Significant hits were taken then into a second phase for validation. To validate our findings we evaluated the performance of behavioral values predicted for breeds not included in the discovery phase. The expectation was to be able to predict behavioral traits from a few markers. Since this data uses publicly available and previously published data, no additional ethics committee approvals were required.

### Discovery behavioral phenotypes

C-BARQ phenotype values and distributions for aggression and fear variables were published for the top 30 most popular breeds of the AKC [21]. The phenotypes for each trait used in this study are provided in Additional file 3: Table S2 (refer to publication for distributions [21]). We refer to this collection of behavioral phenotypes as “C-BARQ phenotypes”. This C-BARQ dataset is a collection of owner reported behavioral data of AKC registered dogs. Only the breeds for which SNP data (see SNP datasets section) were available were included in the analysis; therefore a total of 6,818 subjects were used to determine the phenotypical values. C-BARQ data decomposes aggression into 4 classes: stranger-directed aggression (towards unfamiliar humans), dog-directed aggression (towards unfamiliar dogs), owner-directed aggression and Dog rivalry (towards familiar humans and dogs, respectively). In a similar way C-BARQ data decomposes fear into 5 classes: stranger-oriented fear (towards unfamiliar humans), dog-oriented fear (towards unfamiliar dogs), nonsocial fear (towards environmental phenomena), separation-related anxiety (being left alone by the owner) and touch sensitivity.

### Validation behavioral phenotypes

To validate the hits mapped using the discovery data, we inferred predictions based on the markers detected for the breeds from the Vaysse dataset [15] that were not included in the top 30 most popular breeds [21]. Our predicted C-BARQ values for 18 dog breeds (see Phenotype prediction analysis section) were compared to C-BARQ phenotypes calculated from the data provided in Additional file 3: Table S3 (which includes trait values and distributions). These C-BARQ phenotypical values were obtained from 2,130 subjects of 18 breeds. Only one breed (Greenland Sledge dog) included in the Vaysse dataset had no data available in the C-BARQ database and thus was excluded from the prediction analysis.

### SNP datasets

Two previously published SNP datasets were used in this study. The first dataset contained ~175,000 SNPs on the Illumina CanineHD array; we refer to this dataset as the “Vaysse dataset” [15]. The second dataset contained ~45,000 SNPs on the Affymetrix v.2 Canine array; we refer to this dataset as the “Boyko dataset” [14]. The Vaysse dataset contained a total of 456 subjects representing 30 dog breeds while the Boyko dataset contained 890 subjects representing 80 dog breeds. Since the stereotypic phenotype data was not available for all the breeds included in each of the datasets, only those for which phenotypes were available were kept; therefore, the Vaysse dataset contained 150 subjects from 11 dog breeds (BEAG (Beagle), BULD (English Bulldog), ACKS (American Cocker Spaniel), DASH (Dachshund), DOBP (Doberman Pinscher), GSD- (German Shepherd Dog), GOLD (Golden Retriever), LAB- (Labrador Retriever), SPOO (Standard Poodle), ROTT (Rottweiler), YORK (Yorkshire Terrier)) while the Boyko dataset contained 327 subjects from 29 dog breeds (AUSS (Australian Shepherd), BEAG, BOST (Boston Terrier), BULD, CKCS (Cavalier King Charles Spaniel), CHIH (Chihuahua), ACKS, DASH, DOBP, ESSP (English Springer Spaniel), FBUL (French Bulldog), GSD-, GSHP (German Shorthaired Pointer), GOLD, DANE (Great Dane), HAVA (Havanese), LAB-, MAST (English Mastiff), PEMB (Pembroke Welsh Corgi), POM- (Pomeranian), SPOO, TPOO (Toy Poodle), PUG- (Pug), ROTT, SSHP (Shetland Sheepdog), SHIH (Shih Tzu), HUSK (Siberian Husky), YORK). All dog breeds included in the Vaysse dataset used were also included in the Boyko dataset. Since the Vaysse dataset has higher resolution and cleaner signal as compared to the Boyko dataset (see original publications for more details) we designated the Vaysse dataset as our main discovery dataset while the Boyko dataset would be used for further validation of the findings detected on the Vaysse dataset. Both datasets are independent of each other and no subjects are shared between them.

### Genomewide association analysis and mapping and PCA analysis

The preparation of datasets and subject removal were carried out in PLINK v1.07 [72]. Principal component analysis evaluation was performed on SAS v.9.3 for each dataset separately on the C-BARQ values for the breeds included in the discovery phase (see previous subsection). All association analysis were performed on GEMMA v.0.94.1 [27]. Population structure was removed by using the centered relatedness matrix correction; the association tests were performed using the univariate linear mixed model using the likelihood ratio test. Genomewide significance was declared for the Vaysse dataset for a *P*-value equal or less than 1 × 10^−8^; for the Boyko dataset, genomewide significance was declared for a *P*-value equal or less than 1 × 10^−5^. GEMMA was run on the Ohio Supercomputer Center’s Oakley cluster (www.osc.edu) for faster processing. To avoid irreproducibility issues, no dataset trimming or LD clustering were performed on the SNP data. All Manhattan plots were generated by SAS v.9.3 from GEMMA outputs. Genomewide significant hits were mapped on the UCSC Genome Browser [73] but coordinates were lifted to take advantage of the enhanced annotation available from the Broad Institute CanFam3 Improved Annotation Data V.1 [74] since the original SNP coordinates provided were CanFam2.

### Phenotype prediction analysis

The full description of the methods and results of the prediction modeling are given in Additional file 1. In brief, allele frequencies for the top significant hit for all dog breeds used in the discovery phase and dog breeds not included in the discovery phase (prediction phase breeds) were calculated using PLINK v1.07. Each of the significant markers’ allele frequencies were linearly regressed using a stepwise forward selection method based on an inclusion/exclusion alpha cutoff of ≤ 0.05 excluding the intercept. All statistical modeling was performed on SAS v9.3.

### Probabilistic haplotype analysis of genomewide significant hits

LD blocks of adjacent top significant hits and haplotype determination were evaluated by Haploview v.4.2 [75]. Only top hits were included in the haplotype determination. For hits within the X chromosome all subjects were deemed as females since the Vaysse dataset is not annotated by sex.

### Direct haplotype phasing analysis

We used the top GWA marker for each trait (or for arbitrarily chosen control regions) to segregate individuals within each breed by their carrier status: heterozygous and homozygous. Continuing the analysis within breeds, the homozygotes for alleles A and B were analyzed further. To construct the largest common phased haplotype within a breed, we defined the boundaries by evaluating each SNP upstream and downstream, and keeping only SNP markers that have an allele frequency of at least 0.95. The largest such interval for each allele at the peak SNP was called a phased-haplotype block. In this work we only include within-breed phased-haplotype blocks if they were present in four or more dogs. Allele frequency calculations and data analyses were performed in PLINK v.1.07 [72].

### *S*_*i*_/*D*_*i*_ blocks and gene annotation

We used *S*_*i*_ and *D*_*i*_ data previously published by Vaysse et al. using the same genotype data [15]. This information is available as supplementary material at http://dogs.genouest.org/SWEEP.dir/Supplemental.html. We converted all SNP positions from the original CanFam2 assembly to CanFam3.1 to take advantage of the Broad Institute’s CanFam3 Improved Annotation Data V.1 [74]. The improved gene annotation is available as a public track on the UCSC genome browser.

## Additional files

Additional file 1: Predictive modeling methods and results. (PDF 340 kb) Additional file 2: Figure S1. Summary of prediction equation. **Figure S2.** Mouse *Gnat3* is highly expressed in the Amygdala and Piriform area. (PDF 542 kb) Additional file 3: Table S1. C-BARQ fear and aggression trait descriptions (numbers of questionnaire items in parentheses). **Table S2.** C-BARQ behavioral phenotypes used in the discovery analysis. **Table S3.** C-BARQ behavioral phenotypes used to test predictive model. (PDF 480 kb)
