# Supplementary material for: Genetic mapping of canine fear and aggression
Source: BMC Genomics. 2016 Aug 8;17:572. doi: 10.1186/s12864-016-2936-3 (PMC4977763; doi:10.1186/s12864-016-2936-3)
Supplement: Additional file 1: — Predictive modeling methods and results. (PDF 340 kb) [file 12864_2016_2936_MOESM1_ESM.pdf]

Supplementary Text: Predictive modeling methods and results

Results: Prediction modeling

We next tested the C-BARQ instrument for internal consistency of the behavioral data. Specifically, we wanted to know if the behavioral data is robust as well as qualitatively and quantitatively consistent across breeds. Alternative possibilities include low signal to noise ratios, biases due to human breed-ownership behaviors, and biases related to dog status or behavior that affect participation in C-BARQ study. This analysis does not require one to assume that a trait classified as fear or aggression is necessarily so (or that those can be sub classified in biologically meaningful ways); it is only to test whether the same classification in one group of breeds is the same as that in another group.

Our most robust discovery and confirmation candidates presented above are those four loci significantly-associated with C-BARQ fear and aggression traits in two independent datasets (from different genotyping platforms applied to different breed cohorts): chr10:11169956, chr15:44258017, chr18:23260370, chr18:23298242 and chrX:105245495, chrX:105770058 and chrX:105877339. We used those markers to design a phenotype prediction model that could be applied to the breeds for which C-BARQ behavioral data exists, but has not been published previously. The model was created using our results for the published C-BARQ data for the subset of the top 30 American Kennel Club breeds [1] for which we had genotype data. It was implemented on all other breeds for which we have genotypes within Vaysse's dataset; this was done without prior knowledge of the C-BARQ breed stereotypes that were being predicted. We thus predicted fear and aggression for 18 breeds with existing, but previously unpublished, C-BARQ data. We used a model design of multiple linear regressions with a stepwise forward selection method (Fig. S1). The discovery breeds' allele frequencies at each of the four loci were used as primary data. We allowed the selection method to keep only significant predictors by trait. Then we used the beta coefficients estimated for the predictors remaining in the model, along with the allele frequencies of the prediction phase breeds, to estimate their breed-predicted value for that trait.

Across all prediction models (Fig. S1), the hit on chr10 was never significant and was thus never included. Chr15 was removed by the method for all traits except for owner-directed aggression and dog rivalry, for which it was the only significant predictor. This evidence suggests small dog size is highly correlated with these types of aggression as previously suggested [2], but it is not a strong predictor of other types of fear and aggression. Chr18 hits remained significant in all other traits, with the exception of owner-directed aggression and dog rivalry. In all models, both chr18 hits (chr18:23260370 and chr18:23298242) were included at the beginning of the selection process but only one or the other remained. These two markers are partially correlated and we assume the detection of one or the other is due to the ancestral haplotype diverging across breeds. The chr18 hits were the only significant predictors for dog-oriented fear, dog-directed aggression, nonsocial fear and touch sensitivity. The chrX hits were significant predictors for stranger-oriented fear, stranger-directed aggression and separation-

related anxiety. As with chr18, all three chrX hits detected across all analyses (chrX:105245495, chrX:105770058 and chrX:105877339) were included at the beginning of the selection process, but only one remained for each trait. These markers are very highly correlated and therefore their inclusion in the model can be interchangeable. R-square values for all predictions range from 0.54 to 0.9. We observed that beta coefficients for the chr18 hits are always positive and have values double in size to the beta coefficients for the chrX hit, which is always negative (Fig. S1). Our prediction models suggest that chr18 and X have an additive effect on fear and aggression variability. More precisely, our data suggests that the most common chr18 haplotype across dog breeds is protective from some types of fear/aggression (Fig. 4). In contrast, one of the two common haplotypes on chrX (Fig. 4) increases risk.

To evaluate the performance of our prediction models, we calculated predicted values for all breeds not used in the discovery and confirmation phases and compared them to the values obtained from the raw unreleased C-BARQ data (by calculating the difference between them). To determine whether the performance of our prediction is superior to random chance, we first estimated the chance of randomly making a successful prediction within a 95% confidence interval for all of the observed values. Since the sample sizes for each breed is different, the random chance of making a successful prediction within a 95% confidence is also different. The median chance for making a successful prediction by chance across all breeds and traits is 5.82%, ranging from 1.1% to 30.28%. Next, we calculated the cumulative probability of all random chances. We estimated that 11 out of the 162 values predicted would be expected by chance. Lastly, we evaluated if the difference between the observed and predicted values is inside or outside of the confidence interval, and declared a success for every case where our predicted value was inside. Our total number of successful predictions was 95 out of 164 or 58% which is significantly better than random chance ($p<0.0001$). We propose that this validates the internal consistency of C-BARQ behavioral data across breeds. A success and failure matrix that summarizes our predictions is displayed in Figure 5. Our predictions are not skewed in a single direction across traits and breeds. However, our prediction efficiency is better for some traits and breeds than others. That is consistent with the possibilities that other variants are involved, and that those are not uniformly associated with those traits across many or most breeds.

Methods: Phenotype Prediction Analysis

Significant hits detected across the Vaysse and Boyko datasets for each trait in the discovery phase were further evaluated for their predictive performance. For this part, allele frequencies for the top significant hit for all dog breeds used in the discovery phase and dog breeds not included in the discovery phase (prediction phase breeds) were calculated using PLINK v1.07. Each of the significant markers' allele frequencies were linearly regressed using a stepwise forward selection method based on an inclusion/exclusion alpha cutoff of ≤ 0.05 excluding the intercept. All statistical modeling was performed on SAS v9.3. For each iteration, the selection process tested if any of the allele frequency markers would significantly contribute to the model in terms of P-value significance; if the marker became non-significant after the addition of another marker to

the model then it was removed from the model. The final iteration contained all markers that were below the alpha inclusion/exclusion criteria. Once the equation parameters were estimated, predictions were made using the beta coefficients for each marker that remained in the model with the allele frequencies obtained from the prediction phase breeds. By this method, a prediction value for each of the traits for every breed evaluated was generated.

To evaluate the performance of the aggression and fear predictions made, we calculated mean C-BARQ values observed from the unreleased raw database and calculated 95% confidence intervals for each. Sample size and standard errors used to estimate each of the confidence intervals corresponded to the sample size/standard errors of each breed from the unreleased raw dataset for the respective trait. Since the C-BARQ values are contained within a fixed scale that goes from 0 to 4, a uniform distribution was assumed to calculate the cumulative probability associated to each confidence interval range. With this, it was possible to estimate the chance of making a correct prediction by a random guess. We calculated the difference between the observed and our predicted value and, if the difference was contained within the confidence interval of the observed value we considered the prediction a success, while in the case where the predicted value was outside of the confidence interval, it was considered a failure. To determine if the performance of our prediction outcome was superior to a random guess outcome, we tested using a one-tailed Fishers Exact test if the success /failure counts of our prediction outcome were superior to the random guess outcome. Since our models were generated independent of each other, we did not explore the prediction efficiency scenario where a trait is evaluated given that another trait or traits estimates are known. In summary, we evaluated if the chance of correctly predicting the set of values by random guessing was comparable to the predictions made using our models based on the genomewide significant markers for each trait.

References

1. Serpell, J. and D. Duffy, *Dog Breeds and Their Behavior*, in *Domestic Dog Cognition and Behavior*, A. Horowitz, Editor. 2014, Springer Berlin Heidelberg. p. 31-57.
2. McGreevy, P.D., et al., *Dog Behavior Co-Varies with Height, Bodyweight and Skull Shape.* PLoS ONE, 2013. **8**(12): p. e80529.
