# Supplementary material for: Genetic mapping of canine fear and aggression
Source: BMC Genomics. 2016 Aug 8;17:572. doi: 10.1186/s12864-016-2936-3 (PMC4977763; doi:10.1186/s12864-016-2936-3)
Supplement: Additional file 2: Figure S1. — Summary of prediction equation. Figure S2. Mouse Gnat3 is highly expressed in the Amygdala and Piriform area. (PDF 542 kb) [file 12864_2016_2936_MOESM2_ESM.pdf]

A

$$\boldsymbol{Y} = \mu + \beta1(\mathbf{chrA}:MAF) + \beta2(\mathbf{chrB}:MAF) + \ldots$$

B

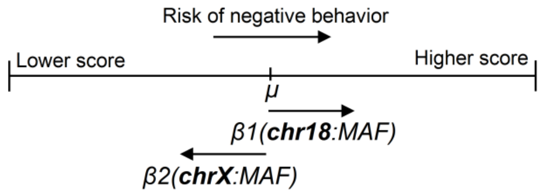

C

Stranger Aggression Score *= 0.6857 + 0.73785(**chr18**:MAF) -0.24217(**chrX**:MAF)*

Dog Aggression Score *= 0.91511 + 0.74044(**chr18**:MAF)*

Owner Aggression Score *= 0.062179+ 0.25121(**chr15**:MAF)*

Rivalry Score *= 0.42109 + 0.353678(**chr15**:MAF)*

Stranger Fear Score *= 0.63331 + 0.80863(**chr18**:MAF) -0.29395(**chrX**:MAF)*

Dog Fear Score *= 0.66267 + 0.58023(**chr18**:MAF)*

Nonsocial Fear Score *= 0.67135 + 0.52984(**chr18**:MAF)*

Separation Related Anxiety Score *= 0.6068 + 0.39148(**chr18**:MAF) -0.23978(**chrX**:MAF)*

Touch Sensitivity Score *= 0.5625 + 0.62219(**chr18**:MAF)*

Fig. S1. Summary of prediction equation. (A) Generalized equation of the model used for the prediction of behavioral traits. MAF corresponds to the Major Allele Frequency of the SNP marker evaluated. (B) Interpretation example of the equation parameters for the prediction of negative behavior. μ sets the overall mean, β1(chr18:MAF) increases the risk while β2(chrX:MAF) decreases the risk. Therefore, a dog breed with high risk for negative behavior has a high chr18:MAF and a low chrX:MAF; on the other side, a dog breed with low risk for negative behavior has a low chr18:MAF and a high chrX:MAF. This specific example applies directly to Stranger-directed aggression, Stranger-oriented fear and Separation-related anxiety. (C) Regression equations for each of the nine C-BARQ behavioral traits.

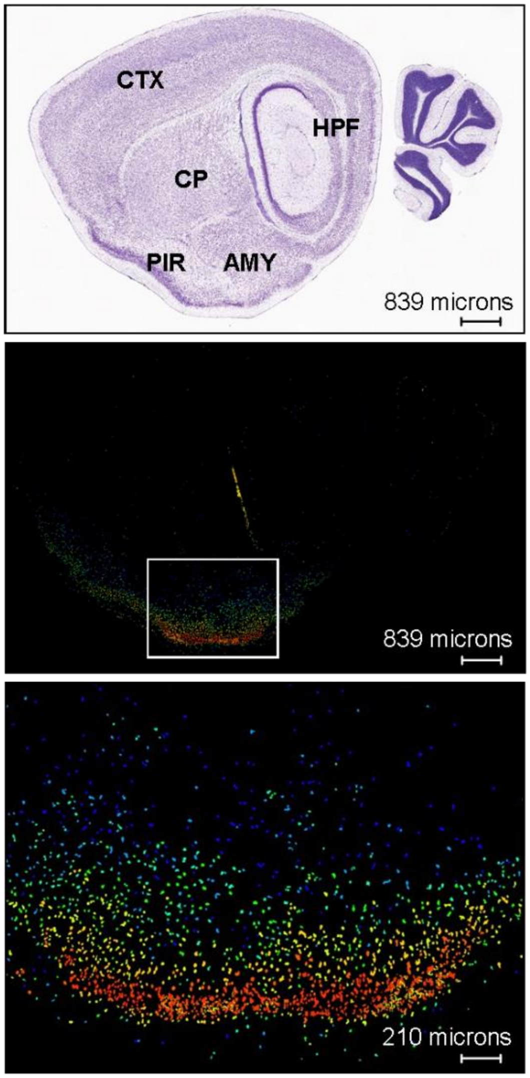

Fig. S2. Mouse *Gnat3* is highly expressed in the Amygdala and Piriform area. Analysis of the Allen Brain Atlas [1] of *in situ* mRNA hybridization data revealed the highest levels of *Gnat3* mRNA expression in the adult mouse to be in the Amygdala and Piriform area (AMY and PYR). The top image is Nissl staining and the bottom two are *in situ* hybridization; signal intensity is represented in a blue (low) to red (high) color spectrum (Atlas Gnat3 Image 1 of 19). The strongest signal is in layer 2 of the Cortical amygdalar area and of the Piriform area. The closeup image at the bottom shows two regions where the most intense color corresponds to the PIR layer 2 (left area of solid red cells) and COA layer 2 (right area of solid red cells); these are separated by an area of intermediate signal strength (orange and yellow cells). The other signal above and perpendicular to AMY/PIR corresponds to the choroid plexus of the lateral ventricle. Another brain area with robust signal is the pons, specifically, in the Lateral reticular nucleus (mixture of red and orange-yellow cells), Paragigantocellular reticular nucleus, lateral part (yellow and blue cells), and the Facial motor nucleus (yellow and blue cells) (see Atlas Images 10 and 13; data not shown). There is also weak to moderate signal in the mitral layer of the olfactory bulb. The

Atlas series has uneven results, with most sections having very low signal. It is thus possible that some brain areas with detectable expression were missed in these experiments. However, the positive pattern shown in the closeup here is not suggestive of non-specific binding. For support of the image shown here, see also Atlas Image 5.

References

1. Lein, E.S., et al., *Genome-wide atlas of gene expression in the adult mouse brain.* Nature, 2007. **445**(7124): p. 168-176.
