# Supplementary material for: Genetic mapping of canine fear and aggression
Source: BMC Genomics. 2016 Aug 8;17:572. doi: 10.1186/s12864-016-2936-3 (PMC4977763; doi:10.1186/s12864-016-2936-3)
Supplement: Additional file 3: Table S1. — C-BARQ fear and aggression trait descriptions (numbers of questionnaire items in parentheses). Table S2. C-BARQ behavioral phenotypes used in the discovery analysis. Table S3. C-BARQ behavioral phenotypes used to test predictive model. (PDF 480 kb) [file 12864_2016_2936_MOESM3_ESM.pdf]

	C-BARQ Trait	C-BARQ Trait Description[1] (number of questionnaire items)
Aggression	Stranger-directed	Severity of threatening or aggressive responses to strangers approaching or invading the dog's or owner's personal space, territory, or home range (10)
	Dog-directed	Severity of threatening or aggressive responses when approached directly by unfamiliar dogs (4)
	Owner-directed	Severity of threatening or aggressive responses to the owner or other members of the household when challenged, manhandled, stared at, stepped over, or when approached while in possession of food or objects (8)
	Dog Rivalry	Severity of aggressive or threatening responses to other familiar dogs in the household (4)
Fear	Stranger-oriented	Severity of fearful or wary responses when approached directly by strange or unfamiliar people (4)
	Dog-oriented	Severity of fearful or wary responses when approached directly by unfamiliar dogs (4)
	Nonsocial	Severity of fearful or wary responses to sudden or loud noises, traffic, and unfamiliar objects and situations (6)
	Separation-related behavior (anxiety)	Frequency of vocalizing and/or destructive behavior when separated from the owner, including autonomic signs of anxiety—restlessness, loss of appetite, trembling, and excessive salivation (8)
	Touch Sensitivity	Severity of fearful or wary responses to potentially painful or uncomfortable procedures, including bathing, grooming, nail-clipping, and veterinary examinations (4)

Table S1. C-BARQ fear and aggression trait descriptions (numbers of questionnaire items in parentheses)

Breed	Breed	Size(Kg)	AgrStra	AgrDog	AgrOwn	AgrRiv	FearStra	FearDog	FearNon	FearSep
Australian Shepherd	AUSS	25	0.757	1.056	0.071	0.557	0.685	0.721	0.762	0.423
Beagle	BEAG	10	0.584	0.977	0.406	0.846	0.669	1.035	0.991	0.966
Boston Terrier	BOST	8	0.522	1.186	0.097	0.667	0.465	0.669	0.799	0.539
Boxer	BOX-	29	0.640	1.223	0.083	0.495	0.549	0.606	0.777	0.608
Bulldog	BULL	24	0.427	0.950	0.209	0.773	0.470	0.779	0.774	0.417
Cavalier King Charles	CKCS	6	0.342	0.376	0.127	0.505	0.575	0.784	0.596	0.536
Chihuahua	CHIH	2	1.081	1.348	0.342	0.941	1.275	1.260	1.085	0.746
Cocker Spaniel	ACKS	10	0.715	0.945	0.261	0.652	0.836	0.852	0.796	0.699
Dachshund	DASH	7	1.277	1.426	0.292	0.766	1.192	1.139	1.016	0.787
Doberman Pinscher	DOBP	35	0.777	1.018	0.061	0.498	0.533	0.564	0.527	0.458
English Springer Spaniel	ESSP	23	0.544	1.145	0.187	0.604	0.606	0.899	0.571	0.542
French Bulldog	FBUL	11	0.607	1.218	0.373	1.121	0.314	0.852	0.652	0.690
German Shepherd	GSD-	37	0.755	1.279	0.090	0.557	0.559	0.685	0.633	0.498
German Shorthaired Pointer	GSHP	26	0.518	0.852	0.111	0.535	0.455	0.606	0.831	0.661
Golden Retriever	GOLD	32	0.292	0.653	0.115	0.374	0.293	0.611	0.712	0.354
Great Dane	DANE	62	0.614	0.936	0.720	0.451	0.747	0.596	0.721	0.536
Havanese	HAVA	6	0.548	0.695	0.160	0.414	0.695	0.862	0.683	0.517
Labrador Retriever	LAB-	30	0.427	0.679	0.099	0.311	0.350	0.585	0.621	0.429
Mastiff	MAST	82	0.618	0.753	0.074	0.454	0.737	0.591	0.839	0.448
Pembroke Welsh Corgi	PEMB	12	0.489	1.045	0.165	0.729	0.392	0.758	0.928	0.439
Pomeranian	POM-	3	0.706	0.972	0.346	0.784	0.669	0.873	0.777	0.649
Standard Poodle	SPOO	25	0.606	0.872	0.119	0.542	0.549	0.664	0.696	0.542
Toy Poodle	TPOO	4	0.772	0.852	0.335	0.740	0.977	0.998	0.984	0.944
Pug	PUG-	7	0.437	0.679	0.171	0.447	0.392	0.800	0.680	0.702
Rottweiler	ROTT	45	0.657	0.867	0.211	0.630	0.287	0.382	0.480	0.332
Shetland Sheepdog	SSHP	8	0.482	0.622	0.133	0.531	0.873	0.747	0.915	0.448
Shih Tzu	SHIH	6	0.610	0.930	0.386	0.700	0.653	1.024	1.000	0.771
Siberian Husky	HUSK	24	0.118	0.721	0.152	0.575	0.220	0.382	0.361	0.473
Yorkshire Terrier	YORK	3	0.953	1.295	0.329	0.806	1.035	0.988	1.047	0.909

Table S2. C-BARQ behavioral phenotypes used in the discovery analysis.
Breeds highlighted in orange are the only ones included in Vaysse et al. 2011. The full list was included in Boyko et al.2010.
DISCLAIMER: The C-BARQ dataset is continuously being updated as new subjects are added to the database. For future derivations of this work, please contact the C-BARQ team directly for the most updated data.

Breed[1]		Trait	N	Mean	StDev	Var
Belgian Tervuren	BeT	DogAggr	110	0.908	0.814	0.663
Belgian Tervuren	BeT	DogFear	110	0.616	0.821	0.674
Belgian Tervuren	BeT	NonFear	112	0.616	0.646	0.417
Belgian Tervuren	BeT	OwnAggr	112	0.118	0.281	0.079
Belgian Tervuren	BeT	RivalryAg	107	0.635	0.632	0.399
Belgian Tervuren	BeT	SepFear	112	0.364	0.493	0.243
Belgian Tervuren	BeT	StrAggr	112	0.64	0.542	0.293
Belgian Tervuren	BeT	StrFear	111	0.632	0.889	0.791
Belgian Tervuren	BeT	TouchFear	111	0.501	0.48	0.231
Bernese Mountain Dog	BMD	DogAggr	195	0.506	0.676	0.456
Bernese Mountain Dog	BMD	DogFear	194	0.5	0.689	0.474
Bernese Mountain Dog	BMD	NonFear	195	0.622	0.678	0.46
Bernese Mountain Dog	BMD	OwnAggr	195	0.061	0.195	0.038
Bernese Mountain Dog	BMD	RivalryAg	185	0.25	0.544	0.296
Bernese Mountain Dog	BMD	SepFear	194	0.261	0.403	0.162
Bernese Mountain Dog	BMD	StrAggr	194	0.349	0.397	0.158
Bernese Mountain Dog	BMD	StrFear	196	0.619	0.905	0.818
Bernese Mountain Dog	BMD	TouchFear	193	0.392	0.469	0.22
Border Collie	BoC	DogAggr	640	1.068	0.99	0.98
Border Collie	BoC	DogFear	649	0.846	0.863	0.746
Border Collie	BoC	NonFear	650	0.965	0.792	0.627
Border Collie	BoC	OwnAggr	659	0.135	0.362	0.131
Border Collie	BoC	RivalryAg	588	0.667	0.81	0.656
Border Collie	BoC	SepFear	661	0.472	0.615	0.378
Border Collie	BoC	StrAggr	649	0.596	0.678	0.46
Border Collie	BoC	StrFear	647	0.731	0.94	0.884
Border Collie	BoC	TouchFear	643	0.75	0.79	0.624
Border Terrier	BoT	DogAggr	70	1.289	1.125	1.265
Border Terrier	BoT	DogFear	72	0.981	0.932	0.868
Border Terrier	BoT	NonFear	70	0.716	0.677	0.459
Border Terrier	BoT	OwnAggr	72	0.097	0.209	0.044
Border Terrier	BoT	RivalryAg	60	0.281	0.633	0.401
Border Terrier	BoT	SepFear	2	0.52	0.532	0.283
Border Terrier	BoT	StrAggr	71	0.405	0.462	0.214
Border Terrier	BoT	StrFear	72	0.524	0.845	0.713
Border Terrier	BoT	TouchFear	71	0.72	0.686	0.47
Brittany Spaniel	BrS	DogAggr	111	0.673	0.778	0.606
Brittany Spaniel	BrS	DogFear	109	0.664	0.764	0.583
Brittany Spaniel	BrS	NonFear	112	0.572	0.498	0.248
Brittany Spaniel	BrS	OwnAggr	116	0.094	0.249	0.062
Brittany Spaniel	BrS	RivalryAg	97	0.353	0.509	0.259
Brittany Spaniel	BrS	SepFear	115	0.661	0.617	0.381
Brittany Spaniel	BrS	StrAggr	115	0.438	0.481	0.232
Brittany Spaniel	BrS	StrFear	116	0.46	0.672	0.451

Brittany Spaniel	BrS	TouchFear	116	0.646	0.673	0.453
Norwegian Elkhound	Elk	DogAggr	33	0.725	0.887	0.787
Norwegian Elkhound	Elk	DogFear	33	0.606	0.862	0.742
Norwegian Elkhound	Elk	NonFear	33	0.631	0.644	0.414
Norwegian Elkhound	Elk	OwnAggr	33	0.225	0.518	0.268
Norwegian Elkhound	Elk	RivalryAg	27	0.241	0.329	0.108
Norwegian Elkhound	Elk	SepFear	33	0.331	0.494	0.244
Norwegian Elkhound	Elk	StrAggr	33	0.605	0.738	0.544
Norwegian Elkhound	Elk	StrFear	33	0.318	0.567	0.321
Norwegian Elkhound	Elk	TouchFear	32	0.69	0.695	0.483
English Setter	ESt	DogAggr	76	0.602	0.827	0.684
English Setter	ESt	DogFear	74	0.72	0.877	0.77
English Setter	ESt	NonFear	76	0.49	0.562	0.316
English Setter	ESt	OwnAggr	78	0.115	0.317	0.101
English Setter	ESt	RivalryAg	67	0.578	0.722	0.521
English Setter	ESt	SepFear	78	0.423	0.577	0.333
English Setter	ESt	StrAggr	78	0.316	0.345	0.119
English Setter	ESt	StrFear	78	0.327	0.538	0.289
English Setter	ESt	TouchFear	75	0.421	0.588	0.346
Eurasier	Eur	DogAggr	45	0.461	0.538	0.29
Eurasier	Eur	DogFear	48	0.293	0.323	0.105
Eurasier	Eur	NonFear	48	0.453	0.42	0.176
Eurasier	Eur	OwnAggr	47	0.118	0.358	0.128
Eurasier	Eur	RivalryAg	44	0.47	0.718	0.515
Eurasier	Eur	SepFear	48	0.271	0.435	0.189
Eurasier	Eur	StrAggr	48	0.382	0.474	0.225
Eurasier	Eur	StrFear	45	0.517	0.728	0.53
Eurasier	Eur	TouchFear	47	0.576	0.568	0.322
Finnish Spitz	FSp	DogAggr	10	1.067	0.7	0.489
Finnish Spitz	FSp	DogFear	10	0.708	0.429	0.184
Finnish Spitz	FSp	NonFear	10	0.92	0.658	0.433
Finnish Spitz	FSp	OwnAggr	10	0.101	0.277	0.077
Finnish Spitz	FSp	RivalryAg	7	1.214	0.847	0.717
Finnish Spitz	FSp	SepFear	10	0.313	0.531	0.282
Finnish Spitz	FSp	StrAggr	10	0.35	0.409	0.167
Finnish Spitz	FSp	StrFear	10	0.5	0.874	0.764
Finnish Spitz	FSp	TouchFear	10	0.7	0.55	0.303
Gordon Setter	GoS	DogAggr	27	0.784	0.784	0.614
Gordon Setter	GoS	DogFear	27	0.657	0.896	0.804
Gordon Setter	GoS	NonFear	27	0.618	0.556	0.309
Gordon Setter	GoS	OwnAggr	27	0.061	0.107	0.011
Gordon Setter	GoS	RivalryAg	26	0.635	0.686	0.471
Gordon Setter	GoS	SepFear	27	0.482	0.717	0.514
Gordon Setter	GoS	StrAggr	26	0.45	0.502	0.252
Gordon Setter	GoS	StrFear	27	0.444	0.864	0.747

Gordon Setter	GoS	TouchFear	27	0.521	0.528	0.278
Greyhound	Gry	DogAggr	138	0.536	0.798	0.636
Greyhound	Gry	DogFear	139	0.758	0.864	0.747
Greyhound	Gry	NonFear	141	1.007	0.849	0.721
Greyhound	Gry	OwnAggr	143	0.132	0.261	0.068
Greyhound	Gry	RivalryAg	120	0.526	0.628	0.394
Greyhound	Gry	SepFear	138	0.629	0.701	0.491
Greyhound	Gry	StrAggr	138	0.138	0.322	0.104
Greyhound	Gry	StrFear	139	0.698	1.077	1.159
Greyhound	Gry	TouchFear	139	0.69	0.759	0.577
Irish Wolfhound	IrW	DogAggr	45	0.541	0.974	0.948
Irish Wolfhound	IrW	DogFear	44	0.472	0.791	0.626
Irish Wolfhound	IrW	NonFear	45	0.66	0.679	0.461
Irish Wolfhound	IrW	OwnAggr	45	0.045	0.094	0.009
Irish Wolfhound	IrW	RivalryAg	38	0.423	0.758	0.574
Irish Wolfhound	IrW	SepFear	45	0.391	0.444	0.197
Irish Wolfhound	IrW	StrAggr	45	0.21	0.235	0.055
Irish Wolfhound	IrW	StrFear	45	0.411	0.745	0.554
Irish Wolfhound	IrW	TouchFear	44	0.511	0.604	0.365
Jack Russel Terrier	JRT	DogAggr	284	1.477	1.142	1.304
Jack Russel Terrier	JRT	DogFear	279	0.826	0.906	0.821
Jack Russel Terrier	JRT	NonFear	285	0.93	0.764	0.584
Jack Russel Terrier	JRT	OwnAggr	291	0.322	0.547	0.299
Jack Russel Terrier	JRT	RivalryAg	242	0.983	0.958	0.918
Jack Russel Terrier	JRT	SepFear	291	0.707	0.668	0.446
Jack Russel Terrier	JRT	StrAggr	286	0.799	0.77	0.592
Jack Russel Terrier	JRT	StrFear	289	0.575	0.837	0.701
Jack Russel Terrier	JRT	TouchFear	279	0.833	0.715	0.511
Newfoundland	NFd	DogAggr	45	0.674	0.782	0.611
Newfoundland	NFd	DogFear	48	0.462	0.653	0.426
Newfoundland	NFd	NonFear	49	0.712	0.643	0.413
Newfoundland	NFd	OwnAggr	49	0.131	0.355	0.126
Newfoundland	NFd	RivalryAg	42	0.448	0.603	0.364
Newfoundland	NFd	SepFear	49	0.455	0.431	0.186
Newfoundland	NFd	StrAggr	49	0.35	0.393	0.154
Newfoundland	NFd	StrFear	49	0.199	0.635	0.404
Newfoundland	NFd	TouchFear	49	0.617	0.611	0.373
Nova Scotia Duck Tolling Retriever	NSD	DogAggr	66	0.607	0.716	0.512
Nova Scotia Duck Tolling Retriever	NSD	DogFear	66	0.525	0.658	0.434
Nova Scotia Duck Tolling Retriever	NSD	NonFear	66	0.701	0.672	0.452
Nova Scotia Duck Tolling Retriever	NSD	OwnAggr	67	0.091	0.208	0.043
Nova Scotia Duck Tolling Retriever	NSD	RivalryAg	62	0.496	0.612	0.374
Nova Scotia Duck Tolling Retriever	NSD	SepFear	67	0.465	0.496	0.246
Nova Scotia Duck Tolling Retriever	NSD	StrAggr	65	0.299	0.354	0.125
Nova Scotia Duck Tolling Retriever	NSD	StrFear	66	0.451	0.603	0.364

Nova Scotia Duck Tolling Retriever	NSD	TouchFear	67	0.657	0.681	0.464
Schipperke	Sci	DogAggr	27	1.016	0.921	0.848
Schipperke	Sci	DogFear	27	0.633	0.681	0.464
Schipperke	Sci	NonFear	27	0.595	0.549	0.301
Schipperke	Sci	OwnAggr	27	0.186	0.347	0.12
Schipperke	Sci	RivalryAg	26	0.667	0.759	0.577
Schipperke	Sci	SepFear	26	0.351	0.385	0.148
Schipperke	Sci	StrAggr	26	0.764	0.717	0.513
Schipperke	Sci	StrFear	26	0.538	0.673	0.453
Schipperke	Sci	TouchFear	26	0.788	0.848	0.719
Chinese Shar-Pei	ShP	DogAggr	37	1.392	1.008	1.016
Chinese Shar-Pei	ShP	DogFear	36	0.701	0.795	0.632
Chinese Shar-Pei	ShP	NonFear	38	0.735	0.76	0.577
Chinese Shar-Pei	ShP	OwnAggr	40	0.238	0.504	0.254
Chinese Shar-Pei	ShP	RivalryAg	26	0.772	0.753	0.567
Chinese Shar-Pei	ShP	SepFear	40	0.699	0.818	0.67
Chinese Shar-Pei	ShP	StrAggr	40	1.029	0.866	0.75
Chinese Shar-Pei	ShP	StrFear	38	0.961	0.907	0.823
Chinese Shar-Pei	ShP	TouchFear	39	1.077	1.081	1.169
Weimaraner	Wei	DogAggr	107	0.805	0.754	0.568
Weimaraner	Wei	DogFear	109	0.878	0.792	0.628
Weimaraner	Wei	NonFear	107	0.612	0.669	0.448
Weimaraner	Wei	OwnAggr	112	0.123	0.401	0.16
Weimaraner	Wei	RivalryAg	97	0.49	0.654	0.427
Weimaraner	Wei	SepFear	111	0.799	0.845	0.714
Weimaraner	Wei	StrAggr	109	0.641	0.683	0.467
Weimaraner	Wei	StrFear	111	0.585	0.776	0.602
Weimaraner	Wei	TouchFear	109	0.616	0.687	0.473

Table S3. C-BARQ behavioral phenotypes used to test predictive model
[1]Breed abbreviations from Vaysse et al. (*1*)

References

1. A. Vaysse *et al.*, Identification of Genomic Regions Associated with Phenotypic Variation between Dog Breeds using Selection Mapping. *PLoS Genet* **7**, e1002316 (2011).
